# Spatial and temporal variations of childhood cancers: Literature review and contribution of the French national registry

**DOI:** 10.1002/cam4.1774

**Published:** 2018-09-19

**Authors:** Stéphanie Goujon, Evangelia Kyrimi, Laure Faure, Sandra Guissou, Denis Hémon, Brigitte Lacour, Jacqueline Clavel

**Affiliations:** ^1^ Epidémiologie des Cancers de l'enfant et de l'adolescent Team (EPICEA) UMR 1153 Epidemiology and Biostatistics Sorbonne Paris Cité Research Center (CRESS) Inserm Villejuif France; ^2^ Sorbonne Paris Cité Paris Descartes University Paris France; ^3^ French National Registry of Childhood Hematological Malignancies (RNHE) Villejuif France; ^4^ French National Registry of Childhood Solid Tumors (RNTSE) Faculté de Médecine CHU Nancy Vandoeuvre‐lès‐Nancy France

**Keywords:** cancer/epidemiology, child, child, preschool, cluster analysis, incidence, spatial analysis, time factors

## Abstract

**Background:**

Significant increases in childhood cancer incidence since the 1970s have been consistently reported worldwide, but the persistence of the increase on recent periods is discussed. No conclusion can be drawn concerning the spatial variations of childhood cancer, either. This study is an in‐depth investigation of the spatial and temporal variations of childhood cancer in France. An extensive review of all the studies published since 2000 on those issues is provided.

**Methods:**

The study included 25 877 cases of childhood cancer registered nationwide over 2000‐2014. The spatial heterogeneity (overdispersion, autocorrelation, overall heterogeneity) was tested, on two geographic scales, and two spatial scan methods were used to detect clusters of cases. The annual average percent change (AAPC) in incidence rate was estimated with Poisson regression models, and joinpoint analyses were considered.

**Results:**

Glioma and non‐Hodgkin lymphoma cases exhibited some spatial heterogeneity and two large clusters were detected. Overall, the incidence rate of childhood cancer was stable over 2000‐2014 (AAPC = −0.1% [−0.3%; 0.2%]). A log‐linear positive trend was significantly evidenced for gliomas other than pilocytic astrocytomas (AAPC = 1.8% [0.9%; 2.7%]), with some suggestion of a leveling‐off at the end of the period, while Burkitt lymphoma and germ cell tumor incidence rates decreased (AAPC = −2.2% [−3.8%; −0.5%] and AAPC = −1.9% [−3.4%; −0.3%], respectively). No spatial heterogeneity or significant time variation was evidenced for other cancers.

**Conclusion:**

Several types of childhood cancer displayed some spatial heterogeneity and two large clusters were detected, the origins of which are to be investigated and might include differences in case ascertainment. Overall, the results do not support a sustained increase in incidence rates of childhood cancer in recent years.

## 1 INTRODUCTION

In France, as in other industrialized countries, the annual incidence rate of childhood cancer is about 150 cases per million children, that is, approximately 1700 new cases per year. Childhood cancer registration has been ensured by the national registry of childhood hematological malignancies since 1990 and the national registry of childhood solid tumors since 2000. The two registries constitute the French national registry of childhood cancer (RNCE). The RNCE regularly produces incidence and survival estimates on a national scale^1^, ^2^ and participates in space‐time surveillance of childhood cancer. The RNCE is involved in cluster investigations and surveillance of at‐risk populations in collaboration with local public health agencies, and investigates the temporal and spatial variations of childhood cancer on a national scale.

Spatial and temporal analyses may provide crucial information on the departure from time or space homogeneity of the process of diagnostic, registration, and classification of diseases of interest and on the etiology of those diseases. The presence of spatial or temporal heterogeneity in the incidence rate may have different origins, of which the presence of one or several spatially/temporally varying risk factors or a general tendency of the diseases to cluster. Underreporting and delays in case notification, better registration in certain areas or periods, differences in clinical practices, and improvements in diagnostic techniques and classifications may also lead to spatial or temporal heterogeneity. If, for any reason, a disease shows a spatial or temporal structure, it needs to be accounted for. As a result, investigating the spatial and temporal variations of incidence rate is a prerequisite for any study on a childhood cancer risk factor that shows a particular spatial or temporal structure.

In this study, we reviewed the recent literature on spatial and temporal variations of childhood cancer. A summary of the papers published since 2000 is presented, by cancer group, in the supporting information (Tables S1‐S11). Significant increases in childhood cancer incidence since the 1970s have been consistently reported. However, the studies based on the most recent periods are less conclusive, and the persistence of the increase beyond the end of the 1990s is still an issue (Tables S1‐S10). Spatial variations of childhood acute leukemia (AL), which accounts for one‐third of childhood cancer cases, have been widely investigated, and several studies reported a spatial heterogeneity.^3^ Fewer studies have focused on the spatial variations of other childhood cancers, but, overall, the results do not support strong spatial heterogeneity.

This study is an in‐depth investigation of the spatial and temporal variations of childhood cancer in France, based on more than 25 000 cases diagnosed over a 15‐year period. The results are discussed in the context of the recent literature.

## 2 METHODS

### 2.1 Childhood cancer cases

Data on childhood cancer cases were provided by the RNCE. All the cases diagnosed over the period 2000‐2014 in 0‐ to 14‐year children living in mainland France at the time of diagnosis were included in the study. Children who came in France for treatment but lived abroad at the time of diagnosis did not meet the RNCE criteria. This led to 25 877 cases including nonmalignant and borderline brain tumors. The diagnoses were coded using the International Classification of Diseases for Oncology, third edition (ICD‐O‐3) and classified further into 12 groups using the International Classification of Childhood Cancer, Third revision, ICCC‐3.^4^

### 2.2 Population data

Mainland France is divided into 95 *départements* (median population in 2006: 534 291; IQR: 299 352‐837 990), 1916 living zones (LZ, median population in 2006: 10 316; IQR: 6635‐19 105) and 35 569 municipalities (median population in 2006: 409; IQR 186‐1004). In 2006, the median population density was 82 inhabitants/km² on the *département* scale (IQR = 50‐150), 63 inhabitants/km² on the LZ scale (IQR = 35‐113), and 37 inhabitants/km² (IQR = 18‐86) on the municipality scale.

The French National Institute of Statistic and Economic Studies (INSEE) provided estimates of the population by year of age on the municipality scale for each census year (1999 and each year between 2006 and 2014), and annual age‐specific population estimates for each *département* from 2000 to 2005. On the municipality scale, population counts were derived from census data for the period 2006‐2014; for inter‐census years (2000‐2005), a linear interpolation between 1999 and 2006 of the proportion of population in each municipality relative to its *département* population was applied to annual *département* estimates. Age‐specific population counts in a LZ were calculated as the sum of the estimated populations of its municipalities.

The age‐specific person‐years at risk were estimated by the mid‐year population estimates. The number of childhood cancer cases expected in each *département* and LZ under the hypothesis of homogeneous incidence rates over the whole territory was then calculated by applying the annual age‐specific national incidence rates to their annual person‐years at risk estimates.

### 2.3 Spatial variations

Spatial analyses were conducted on the LZ and *département* scales. As some methods do not address the particular case of isolated units with no neighbor, the LZ located on islands and the Corsica *département* (<1% of the total population) were excluded from the analyses, leaving 1898 LZ and 94 *départements*.

#### 2.3.1 Tests for overall heterogeneity (clustering)

Three methods were used to test for overall spatial heterogeneity in the incidence rate of childhood cancer. The Potthoff and Whittinghill method tests for overdispersion^5^ assuming that, under the hypothesis of overdispersion, the numbers of cases in the geographic units have a negative binomial distribution. The variance to mean ratio is assumed to be equal to 1 + *b*, with *b* > 0, the overdispersion parameter. The variance of the “*b*” parameter depends greatly on the number of units under consideration (Potthoff and Whittinghill). Therefore, we also considered the standardized parameter estimate. Overdispersion can be due to a high variability in counts around the number of cases expected under the Poisson distribution assumption (known as extra‐Poisson variation), but it can also be due to differences in the area specific relative risks. In the latter situation, it may reflect the presence of risk factors in some particular areas. The second method is based on Moran's I spatial autocorrelation statistic.^6^ A null value corresponds to spatial independence between incidence rates, while positive values reflect similarities between incidence rates in neighboring areas, which could be driven by a spatially structured environmental factor. The third method, the Rogerson's test, focuses both on within‐ and between‐area incidence rate variability and thus combines two terms: an autocorrelation term and the common Chi2 statistic of goodness of fit that compares observed and expected local counts.^7^ A significant result can be the reflection of either overdispersion or a spatial dependence between incidence rates in adjacent units.

For autocorrelation terms, geographic units were considered neighboring areas if they shared a common border.

#### 2.3.2 Tests for cluster detection

Two methods based on a moving window that scans the whole country were used to detect clusters of childhood cancer: the spatial scan method^8^ implemented using SaTScan (v.9.4.2) software^9^ and the flexible scan method^10^ implemented using FleXScan (v3.1.2) software.^11^ Both methods, referred to as the SaTScan method and FleXscan method hereafter, build a set of cluster candidates composed of neighboring areas and consider, for each, the likelihood ratio based on the alternative hypothesis that the incidence rate is higher inside than outside the cluster candidate, and the null hypothesis that both incidence rates are equal. The zone that maximizes the likelihood ratio function is defined as the most likely cluster. The SaTScan method creates a set of circular or elliptic cluster candidates while the FleXScan method considers irregularly shaped zones.

The maximum cluster size in both methods was set to 100 LZ or 20 *départements*. For computational reason, a restriction parameter was used for the FleXScan method on the LZ scale so that only cluster candidates made up of units with significant local excesses of cases were considered (local threshold = 0.20).

#### 2.3.3 Significance threshold

In spatial analyses, the *P*‐values of the statistical tests were determined on the basis of simulations. Those simulations were obtained from a multinomial distribution of the total number of observed cases with probabilities proportional to the numbers of expected cases in the geographic units (at least 999 and 9999 simulations for the LZ and *département* scales, respectively).

### 2.4 Time variations

Over the period 2000‐2014, the age distribution of the person‐years at risk was stable (Table S12). Crude incidence rates were thus estimated for all cancers and by diagnostic group. The presence of a log‐linear temporal trend was tested with Poisson regression models with the annual at‐risk person‐years (PY_*i*_, *i* = 2000‐2014) as an offset: Ln(*E*(*O*_*i*_)) = Ln(PY_*i*_) + *α* + β*i with *O*_*i*_ the number of cases diagnosed in year *i*,* α* the intercept and *β* the slope parameter. The average annual percent change (AAPC) was derived from the slope parameter estimate $\hat{\beta }$ as AAPC = (exp($\hat{\beta }$)−1)*100. The degree of temporal overdispersion was estimated by the ratio of the deviance to the number of degrees of freedom. In the event that overdispersion was suspected, a negative binomial regression model was considered. Interactions with age (0‐6 year and 7‐14 year groups) and gender were tested if applicable.

A joinpoint analysis was also considered systematically to allow for piecewise linear variations.^12^ The method was implemented with the Joinpoint Software developed by the SEER program^13^ with the following constraints: at most two joinpoints; at least three observations between two consecutive joinpoints; at least three observations between joinpoints and endpoints. When a nonlinear trend was graphically suggested by the data, we also fitted a generalized additive model with a loess smoothing.

### 2.5 Statistical analyses

The analyses were performed for all childhood cancer and by diagnostic groups and main subgroups. We excluded Langerhans cell histiocytosis cases and the groups of “Other malignant epithelial neoplasms and malignant melanomas” and “Other and unspecified malignant neoplasms” (groups 11 and 12 in the ICCC‐3) because they may be subject to spatial and temporal variations in registration in France. For the central nervous system tumor analyses, three main subgroups were considered: ependymomas and plexus choroid tumors, embryonal CNS tumors, and gliomas. The latter subgroup was split into pilocytic astrocytoma and other glioma groups.

Sensitivity analyses were conducted for the glioma group with a restriction to cases with a histologically confirmed diagnosis.

Two time periods (2000‐2006, 2007‐2014) were considered in order to evaluate the temporal stability of the main clustering results and the stability of the detected clusters. When overdispersion was evidenced significantly on the *département* scale (Potthoff and Whittinghill method), we excluded, in a sensitivity analysis, the *départements* covered by a local cancer registry (adults and children cases), the data of which are regularly crosschecked with the RNCE database. There are 25 local registries in mainland France, 11 of which are site‐specific cancer registries. Some cases, not treated in a hospital unit covered by RNCE active searching, can be identified in that way. Although they represent a small number of cases (<5 cases per year on average, excluding thyroid carcinomas and malignant melanomas), the crosschecking procedure could induce some spatial heterogeneity on the *département* scale.

In situations of overall spatial heterogeneity of incidence rates on the *département* scale, we accounted for it in a temporal sensitivity analysis by considering a *département* specific intercept in the Poisson regression model. An attenuation of the slope parameter could indicate the presence of some *départements* with a higher/lower incidence rate that would contribute both to the spatial heterogeneity and the temporal trend.

## 3 RESULTS

Over the period 2000‐2014, 25 877 tumors were diagnosed in children aged fourteen years or less and living in mainland France at the time of diagnosis, equivalent to an overall age‐standardized incidence rate (world reference) of 155.6 cases/million/y (Table 1).

**Table 1:** Incidence rate of childhood cancer in France over 2000‐2014

Diagnostic groups and subgroups	N	%	Incidence rate (/million/y)		M/F
			Crude	ASR	
Malignant hematopoietic tumors					
Leukemias, myeloproliferative, and myelodysplastic diseases	7447	29	43.5	45.4	1.3
Acute lymphoid leukemias	5854		34.2	35.8	1.3
Acute myeloid leukemias	1078		6.3	6.5	1.1
Lymphomas	2832	11	16.5	15.5	2.0
Hodgkin lymphomas	1262		7.4	6.6	1.4
Burkitt lymphomas	699		4.1	4.0	5.2
Other lymphomas	871		5.1	4.9	1.8
CNS tumors	6359	25	37.1	37.7	1.2
Ependymomas	625		3.7	3.9	1.4
Embryonal CNS tumors	1228		7.2	7.5	1.5
Gliomas	3898		22.8	22.9	1.1
Pilocytic astrocytomas	1409		8.2	8.4	1.1
Other gliomas	2489		14.5	14.6	1.1
Embryonal tumors					
Neuroblastomas	2101	8	12.3	13.9	1.1
Retinoblastomas	742	3	4.3	5.0	1.0
Nephroblastomas	1415	5	8.3	9.3	0.9
Hepatoblastomas	218	1	1.3	1.4	1.4
Malignant bone and soft tissue tumors					
Malignant bone tumors	1225	5	7.2	6.6	1.1
Osteosarcomas	576		3.4	3.1	1.0
Ewing tumors	556		3.2	3.1	1.3
Soft tissue and other extraosseous sarcomas	1654	6	9.7	9.9	1.4
Rhabdomyosarcomas	899		5.3	5.5	1.6
Other soft tissue sarcomas	755		4.4	4.4	1.2
Germ‐cell tumors, trophoblastic tumors, and neoplasms of gonads	836	3	4.9	4.9	0.8
Intracranial and intraspinal GCT	229		1.3	1.2	2.2
Malignant extracranial and extragonadal GCT	263		1.5	1.7	0.6
Malignant gonadal GCT	318		1.9	1.8	0.5
Other malignant epithelial neoplasms and malignant melanomas	869	3	5.1	4.7	0.7
Thyroid carcinomas	369		2.2	2.0	0.5
Malignant melanomas	160		0.9	0.9	1.0
All cancers	25 877	100	151.1	155.6	1.2

N, Number of cases, ASR, Age Standardized incidence Rate (world reference), M/F, sex ratio Male/Female; CNS, Central Nervous System tumor; GCT, Germ Cell Tumor.

### 3.1 Spatial variations

#### 3.1.1 Spatial heterogeneity

Overall, some overdispersion ($\hat{b}$ = 0.38, $\hat{b\_\text{sd}}$ = 2.6) and a significant global heterogeneity of childhood cancer cases was found on the *département* scale, but no significant spatial autocorrelation (Table 2).

**Table 2:** Spatial heterogeneity of childhood cancer incidence on the living zone and *département* scales (France, 2000‐2014)

Diagnostic groups and subgroups	N	n2	Living zone^a^							n2	*Département* b						
			Spatial overdispersion			Spatial autocorrelation		Global heterogeneity			Spatial overdispersion			Spatial autocorrelation		Global heterogeneity	
			$\hat{b}$	$\hat{b\_\text{sd}}$	*P*	*I*	*P*	*R*	*P*		$\hat{b}$	$\hat{b\_\text{sd}}$	*P*	*I*	*P*	*R*	*P*
Malignant hematopoietic tumors																	
Leukemias, myeloproliferative, and myelodysplastic diseases	7447	837	−0.04	−1.2	0.92	0.00	0.40	0.25	0.51	95	−0.10	−0.7	0.73	0.20	0.26	0.02	0.23
Acute lymphoid leukemias	5854	702	−0.06	−1.8	0.96	0.00	0.41	0.31	0.71	95	−0.02	−0.1	0.53	0.25	0.10	0.02	0.08
Acute myeloid leukemias	1078	143	0.00	−0.1	0.51	0.00	0.43	1.84	0.28	91	−0.05	−0.3	0.62	0.10	0.90	0.08	0.53
Lymphomas	2832	381	0.02	0.6	0.23	0.01	0.24	0.75	0.09	95	0.15	1.0	0.15	0.13	0.80	0.04	0.18
Hodgkin lymphomas	1262	174	0.02	0.7	0.25	0.02	0.12	1.67	0.14	92	0.12	0.8	0.21	0.35	<0.01	0.13	0.03
Burkitt lymphomas	699	97	0.00	0.1	0.45	0.02	0.10	3.11	0.09	79	0.24	1.6	0.06	0.19	0.36	0.20	0.07
Other lymphomas	871	124	0.04	1.1	0.14	0.01	0.32	2.39	0.17	86	0.36	2.5	0.01	0.14	0.70	0.17	0.05
CNS tumors	6359	762	0.08	2.5	0.01	−0.01	0.66	0.32	0.18	95	0.45	3.1	<0.01	0.23	0.15	0.03	<0.01
Ependymomas and choroid plexus tumors	625	66	0.03	0.9	0.19	−0.03	0.99	2.91	0.63	78	0.14	0.9	0.18	0.10	0.90	0.07	0.94
Embryonal CNS tumors	1228	165	0.02	0.6	0.21	0.00	0.36	1.45	0.71	91	−0.04	−0.3	0.59	0.22	0.20	0.07	0.48
Gliomas	3898	523	0.10	3.0	<0.01	0.00	0.44	0.58	0.01	95	0.67	4.6	<0.01	0.21	0.25	0.06	<0.01
Pilocytic astrocytomas	1409	207	0.03	0.9	0.18	−0.03	0.98	1.24	0.78	91	0.22	1.5	0.07	0.14	0.69	0.09	0.12
Other gliomas	2489	348	0.08	2.3	0.02	0.01	0.28	0.94	<0.01	95	0.66	4.5	<0.01	0.24	0.13	0.09	<0.01
Embryonal tumors																	
Neuroblastomas	2101	266	−0.02	−0.6	0.74	0.01	0.29	0.83	0.82	93	0.15	1.1	0.15	0.26	0.05	0.06	0.15
Retinoblastomas	742	83	−0.02	−0.6	0.74	0.00	0.53	2.31	0.82	83	0.00	0.0	0.49	0.07	0.98	0.10	0.65
Nephroblastomas	1415	181	0.03	0.8	0.20	0.02	0.06	1.72	<0.01	91	0.11	0.8	0.21	0.13	0.71	0.08	0.21
Hepatoblastomas	218	24	0.08	2.6	0.02	−0.02	0.99	7.81	0.75	49	−0.03	−0.2	0.55	0.12	0.83	0.34	0.66
Malignant bone and soft tissue tumors																	
Malignant bone tumors	1225	169	0.01	0.2	0.41	0.02	0.07	1.75	0.10	91	−0.11	−0.8	0.77	0.19	0.38	0.05	0.80
Osteosarcomas	576	74	0.00	−0.1	0.50	0.02	0.12	3.14	0.67	81	−0.09	−0.6	0.71	0.26	0.07	0.20	0.20
Ewing tumors	556	73	−0.02	−0.5	0.65	0.00	0.49	3.79	0.17	81	−0.04	−0.3	0.59	0.19	0.37	0.10	0.87
Soft tissue and other extraosseous sarcomas	1654	230	0.03	0.9	0.17	−0.02	0.94	1.02	0.88	91	0.11	0.7	0.22	0.22	0.16	0.05	0.50
Rhabdomyosarcomas	899	125	0.01	0.2	0.41	−0.02	0.98	1.94	0.77	87	−0.10	−0.7	0.74	0.15	0.61	0.04	0.97
Other soft tissue sarcomas	755	101	−0.01	−0.2	0.58	−0.02	0.94	2.26	0.83	79	0.09	0.6	0.24	0.26	0.08	0.17	0.11
Germ‐cell tumors, trophoblastic tumors, and neoplasms of gonads	836	116	0.02	0.6	0.25	0.00	0.42	2.11	0.76	86	0.07	0.5	0.31	0.14	0.69	0.10	0.43
Intracranial and intraspinal GCT	229	31	−0.02	−0.6	0.72	−0.01	0.78	7.34	0.78	57	−0.15	−1.0	0.86	0.20	0.29	0.42	0.35
Malignant extracranial and extragonadal GCT	263	29	0.02	0.7	0.22	−0.01	0.63	7.38	0.40	52	0.02	0.1	0.43	0.17	0.49	0.29	0.63
Malignant gonadal GCT	318	37	−0.01	−0.3	0.57	0.04	0.02	5.74	0.60	60	−0.08	−0.5	0.69	0.20	0.31	0.28	0.46
All cancers	25 877	1610	0.03	1.0	0.16	0.00	0.39	0.09	0.02	95	0.38	2.6	0.01	0.18	0.43	0.01	0.05

CNS, Central nervous system; GCT, germ cell tumors.

n2: number of areas with 2 observed cases or more. $\hat{b}$: overdispersion parameter estimate (Potthoff and Whittinghill's test); $\hat{b\_\text{sd}}$: standardized overdispersion parameter estimate $\hat{b}/\hat{\text{sd}}$ with $\hat{\text{sd}}=\sqrt{\frac{2N}{\left(m-1)(N-1\right)}}$ where *m* = total number of geographic units. The Statistical significance level was based on the one‐sided tail probability of the null distribution (1000 Monte Carlo simulations). *I* and *R* refer to Moran's autocorrelation statistic and Rogerson's statistic.

a 1916 LZ for spatial overdispersion, 1898 LZ for spatial autocorrelation and overall heterogeneity (islands excluded).

b 95 *départements* for spatial overdispersion, 94 *départements* for spatial autocorrelation and overall heterogeneity (Corsica excluded).

A significant spatial heterogeneity was evidenced on the *département* scale for the lymphoma subgroups, with overdispersion for non‐Hodgkin lymphomas (NHL) ($\hat{b}$ = 0.24, $\hat{b\_\text{sd}}$ = 1.6 *P* = 0.06 and $\hat{b}$ = 0.36, $\hat{b\_\text{sd}}$ = 2.5, *P* = 0.01 for Burkitt lymphomas and other NHL, respectively) and a significant spatial autocorrelation for Hodgkin lymphomas (*I* = 0.35, *P* < 0.01). With <5 cases per *département*, the results for Burkitt lymphomas and other NHL were not stable by period (not shown).

Overdispersion was also observed for the whole group of gliomas (3898 cases), on both geographic scales ($\hat{b}$ = 0.10, $\hat{b\_\text{sd}}$ = 3.0, *P* < 0.01 and $\hat{b}$ = 0.67, $\hat{b\_\text{sd}}$ = 4.6, *P* < 0.01 on the LZ and *département* scales, respectively). Pilocytic astrocytomas and other gliomas groups were both heterogeneously spatially distributed, and overdispersed on the *département* scale ($\hat{b}$ = 0.22, $\hat{b\_\text{sd}}$ = 1.5, *P* = 0.07 for pilocytic astrocytomas and $\hat{b}$ = 0.66, $\hat{b\_\text{sd}}$ = 4.5, *P* < 0.01 for other gliomas). On the *département* scale, similar results were observed for 2000‐2006 and 2007‐2014 for other gliomas, but the results were not consistent for pilocytic astrocytomas (not shown). Analyses by period on the LZ scale were more limited because of the very small numbers of cases per unit. When the “other glioma” group was restricted to histologically confirmed cases (1702 cases, 68%), the overdispersion parameter estimate was reduced by half on the *département* scale ($\hat{b}$ = 0.31, $\hat{b\_\text{sd}}$ = 2.1, *P* = 0.02, not shown).

A spatial autocorrelation was evidenced on the *département* scale for neuroblastomas (*I* = 0.26, *P* = 0.05). With few contributive units, a small spatial heterogeneity was also observed on the LZ scale for nephroblastomas (autocorrelation and overall heterogeneity), hepatoblastomas (overdispersion), and malignant gonadal germ cell tumors (autocorrelation).

No spatial heterogeneity was evidenced for the other childhood cancer groups (Table 2).

#### 3.1.2 Cluster detection

**Figure 1:**
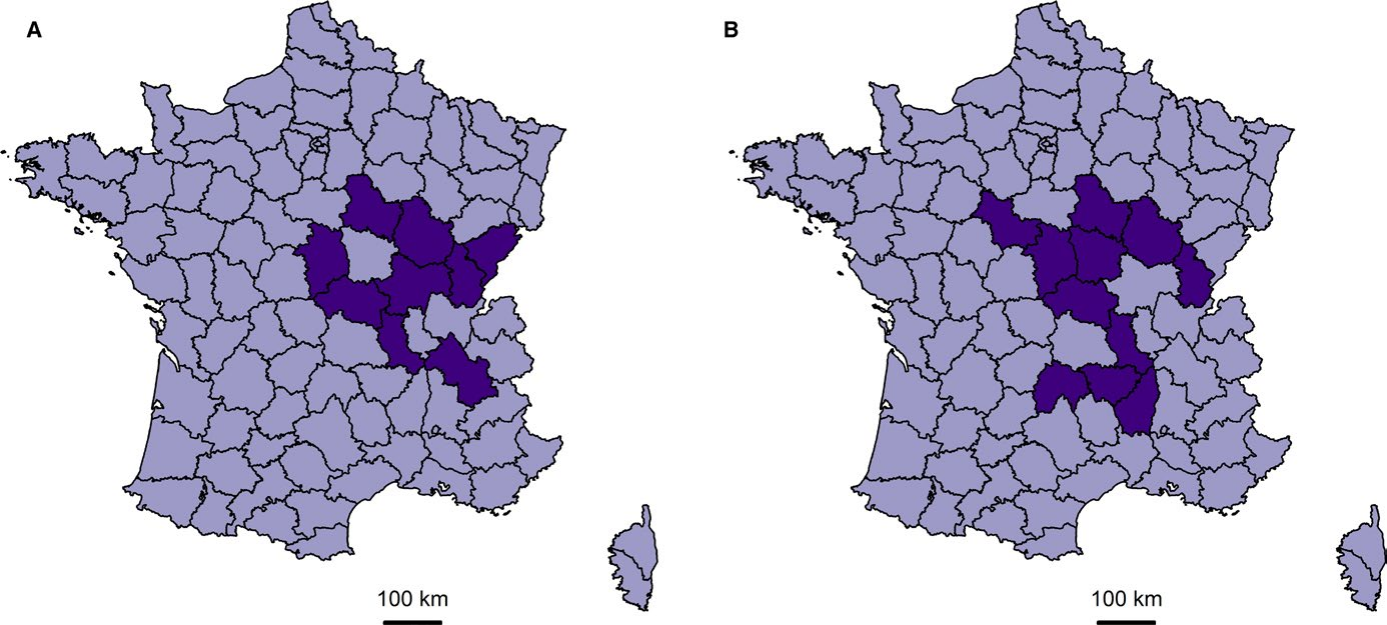
Cluster of lymphomas (A) and non‐Burkitt non‐Hodgkin lymphomas (B) detected over 2000‐2014 with the FleXScan method

A widespread irregularly shaped cluster of lymphomas, located in the Center‐East of France, was found on the *département* scale with the FleXScan method (9 *départements*, with 285 observed cases (*O*) and 216.1 expected cases (*E*), *P* = 0.04) (Table 3>, Figure 1A). A small cluster of non‐Burkitt NHL (5 LZ with *O* = 12, *E* = 1.3, *P* < 0.01) was detected in the North‐West of France, with SaTScan and FleXScan methods, and a large irregularly shaped cluster (11 *départements*,* O* = 85, *E* = 50, *P* = 0.04 with FleXScan) was located in the Center of France, somewhat in the same region as the large lymphoma cluster (Table 3, Figure 1B). A descriptive analysis showed that Burkitt lymphoma cases and other NHL cases were in excess in the all‐lymphoma cluster (*O* = 78 and *E* = 53.1 for Burkitt lymphomas, *O* = 99 and *E* = 66.4 for other NHL). Excluding the cluster area from the clustering analyses did not greatly change the results, as 23% overdispersion was observed for both Burkitt lymphomas and other NHL (not shown).

**Table 3:** Most likely clusters of childhood cancer detected by SaTScan and FleXScan, on the living zone and *département* scales (2000‐2014)

Diagnostic groups and subgroups	N	1898 LZ								94 *Départements*							
		SaTScan				FleXScan				SaTScan				FleXScan			
		Units	*O*	*E*	*P*	Units	*O*	*E*	*P*	Units	*O*	*E*	*P*	Units	*O*	*E*	*P*
Malignant hematopoietic tumors																	
Leukemias, myeloproliferative and myelodysplastic diseases	7447	94	325	262.1	0.93	11	55	28.2	0.49	11	562	490.6	0.55	12	893	774.9	0.12
Acute lymphoid leukemias	5854	51	166	118.7	0.69	13	67	32.5	0.10	17	698	611.0	0.25	12	731	621.2	0.06
Acute myeloid leukemias	1078	7	8	1.2	0.64	6	11	2.4	0.71	7	233	191.9	0.59	11	208	161.8	0.40
Lymphomas	2832	48	132	85.3	0.09	6	17	4.5	0.45	17	431	358.7	0.09	9	285	216.1	0.04
Hodgkin lymphomas	1262	94	84	51.2	0.43	7	13	3.1	0.63	5	69	41.5	0.10	6	79	48.7	0.21
Burkitt l ymphomas	699	4	9	1.0	0.07	5	10	1.2	0.07	19	129	96.8	0.42	12	131	92.0	0.18
Other lymphomas	871	5	12	1.3	<0.01	5	12	1.3	0.01	3	37	18.6	0.15	11	85	50.0	0.04
CNS tumors	6359	97	325	240.7	0.02	23	213	138.7	0.02	11	497	392.9	<0.01	9	478	367.3	<0.01
Ependymomas and choroid plexus tumors	625	5	10	1.3	0.08	2	5	0.4	0.42	6	43	28.2	0.94	13	73	50.3	0.86
Embryonal CNS tumors	1228	12	12	2.7	0.53	3	7	1.1	0.90	17	156	118.6	0.35	12	122	86.3	0.43
Gliomas	3898	57	204	131.9	<0.01	22	136	77.0	0.01	11	337	241.3	<0.01	10	328	225.2	<0.01
Pilocytic astrocytomas	1409	8	11	2.7	0.91	9	28	12.2	0.85	9	243	200.7	0.64	12	202	171.5	0.66
Other gliomas	2489	97	159	95.1	<0.01	33	75	28.5	<0.01	11	230	154.4	<0.01	8	204	129.4	<0.01
Embryonal tumors																	
Neuroblastomas	2101	65	127	83.5	0.22	13	38	16.1	0.31	6	184	137.1	0.10	13	380	309.2	0.12
Retinoblastomas	742	45	33	16.8	0.99	8	17	5.3	0.53	2	49	28.7	0.31	7	143	99.4	0.08
Nephroblastomas	1415	18	14	3.0	0.18	14	24	5.6	0.03	11	270	221.9	0.39	12	301	240.8	0.20
Hepatoblastomas	218	2	3	0.1	0.63	1	2	0.0	0.60	2	7	1.6	0.56	11	36	21.1	0.84
Malignant bone and soft tissue tumors																	
Malignant bone tumors	1225	41	31	13.4	0.60	10	23	8.6	0.68	6	62	37.2	0.16	8	106	70.1	0.18
Osteosarcomas	576	4	6	0.5	0.37	4	6	0.5	0.31	6	125	91.7	0.23	14	181	138.9	0.26
Ewing tumors	556	32	14	3.2	0.25	4	6	0.5	0.23	3	16	5.5	0.22	7	34	16.8	0.40
Soft tissue and other extraosseous sarcomas	1654	4	6	0.6	0.60	8	14	4.3	0.95	7	187	150.6	0.74	12	395	330.4	0.30
Rhabdomyosarcomas	899	2	6	0.7	0.87	2	6	0.7	0.80	9	98	72.7	0.77	12	249	203.0	0.51
Other soft tissue sarcomas	755	60	28	11.9	0.69	6	9	2.0	0.87	12	122	90.4	0.40	10	113	81.0	0.55
Germ‐cell tumors, trophoblastic tumors, and neoplasms of gonads	836	33	24	8.4	0.25	2	7	1.4	0.99	5	106	78.8	0.68	10	166	127.6	0.54
Intracranial and intraspinal GCTs	229	7	4	0.33	0.95	2	2	0.1	0.98	4	12	4.72	0.83	10	23	10.76	0.73
Malignant extracranial and extragonadal GCT	263	5	5	0.35	0.42	1	3	0.1	0.33	5	11	3.69	0.62	13	33	16.15	0.35
Malignant gonadal GCT	318	44	17	4.8	0.28	2	4	0.3	0.72	7	73	53.8	0.89	9	84	60.2	0.81
All cancers	25 877	82	1210	1061.4	0.26	26	720	587.8	0.12	10	1789	1605.2	0.01	10	2519	2278.7	0.01

CNS, Central nervous system; GCT, Germ cell tumors.

N: total number of observed cases (islands excluded). units: number of geographic units (LZ or *département*) included in the most likely cluster zone. *O* and *E*: observed and expected numbers of cases in the most likely cluster zone. *P*: statistical significance threshold after 999 and 9999 Monte Carlo simulations on the LZ and *département* scales, respectively.

**Figure 2:**
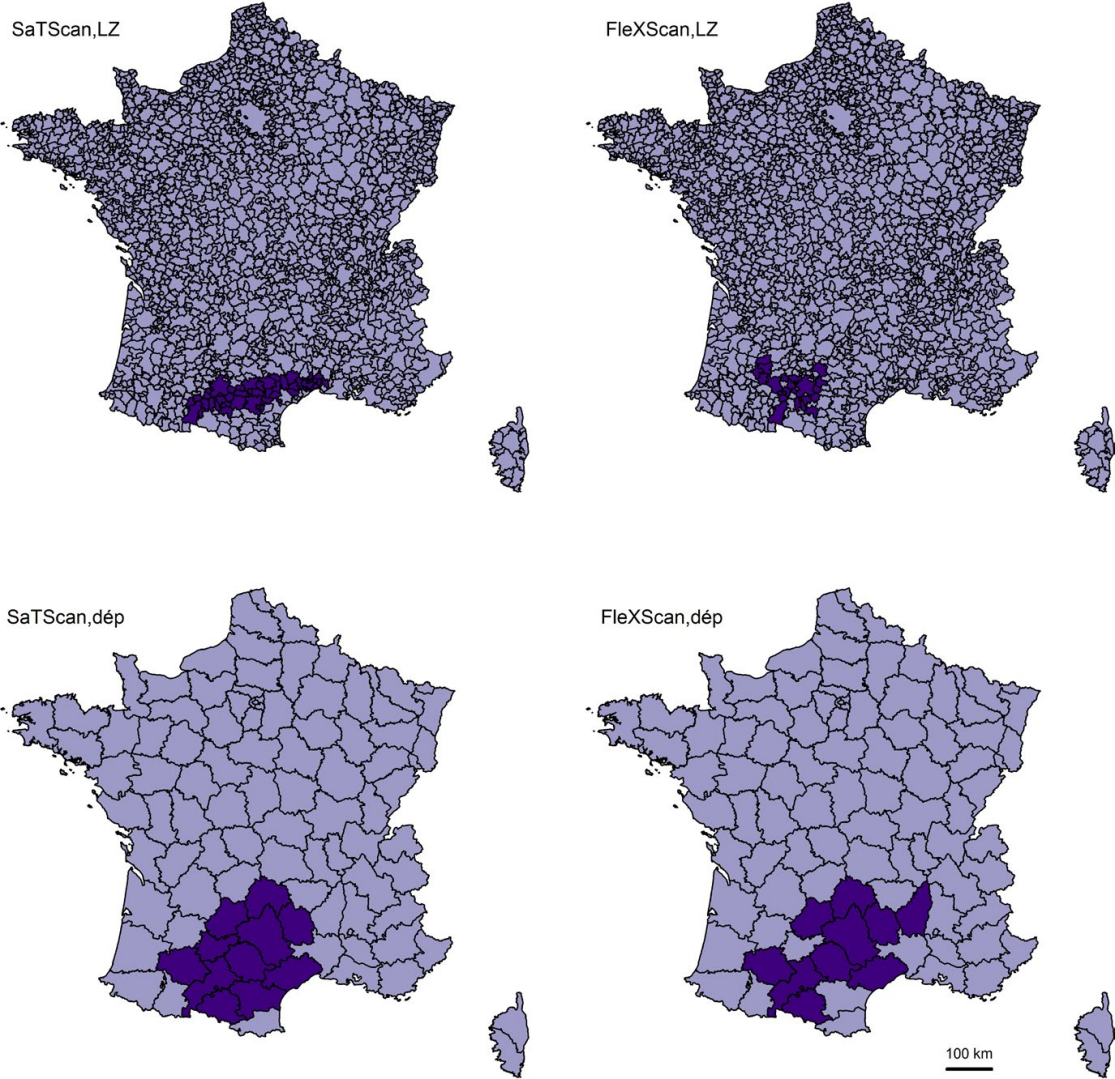
Clusters of gliomas detected by SaTScan and FleXScan methods over 2000‐2014, on the living‐zone (LZ) and *département* (dép) scales

On both geographic scales, both methods detected a cluster of CNS tumors, due to an excess of gliomas cases, in the southern region of France, mainly in the Occitanie region. Highly significant overlapping clusters of gliomas were found in that region (Table 3, Figure 2). The smaller cluster spread over 22 LZ (*O* = 136, *E* = 77.0, *P* < 0.01), while the larger cluster covered 11 *départements* with 337 observed cases and 241.3 expected cases (*P* < 0.01). The cluster areas detected by SaTScan and FleXScan had nine *départements* in common, with 303 observed cases (97 pilocytic astrocytomas and 206 other gliomas) and 206.5 expected cases (74.5 and 132.0 for pilocytic astrocytomas and other gliomas, respectively). Analyses on gliomas other than pilocytic astrocytomas generated quite similar results, with larger cluster areas detected on the LZ scale. Excluding the Occitanie region from clustering analyses (results not shown) did not change the results on the LZ scale, but halved the amount of overdispersion for all gliomas ($\hat{b}$ = 0.38, $\hat{b\_\text{sd}}$ = 2.4, *P* = 0.01) and “other gliomas” ($\hat{b}$ = 0.37, $\hat{b\_\text{sd}}$ = 2.4, *P* = 0.01) on the *département* scale. Interestingly, no significant cluster was detected when the glioma group was restricted to cases with histological confirmation of the diagnosis, neither with SaTScan nor FleXScan (not shown).

A cluster was also detected, only with FleXScan on the LZ scale, for nephroblastomas (14 LZ, *O* = 24, *E* = 5.6, *P* = 0.03).

All the detected clusters were quite stable over time: excesses of cases were observed in the cluster areas, not only over the whole time period 2000‐2014, but also over the periods 2000‐2006 and 2007‐2014, with observed to expected cases ratios of a similar order of magnitude (not shown).

### 3.2 Time variations

Overall, the incidence rate of childhood cancer was stable between 2000 and 2014, with an estimated AAPC of −0.1% [−0.3%; 0.2%] (*P* = 0.69) (Table 4).

**Table 4:** Time variation in the incidence rate of childhood cancer in France over 2000‐2014

Diagnostic groups and subgroups	N^a^	Φ^b^	AAPC (%)	95% CI^c^	*P*‐value
Malignant hematopoietic tumors					
Leukemias, myeloproliferative, and myelodysplastic diseases	7447		0.1	−0.4; 0.7	0.64
Acute lymphoid leukemias	5854		0.4	−0.2; 1.0	0.23
Acute myeloid leukemias	1078		−0.6	−2.0; 0.8	0.38
Lymphomas	2832		−1.1	−1.9; −0.2	0.01
Hodgkin lymphomas	1262		−0.3	−1.6; 0.9	0.60
Burkitt lymphomas	699		−2.2	−3.8; −0.5	0.01
Other lymphomas	871		−1.2	−2.7; 0.3	0.12
CNS tumors	6359		0.4	−0.1; 1.0	0.13
Ependymomas and choroid plexus tumors	625		0.8	−1.0; 2.6	0.39
Embryonal CNS tumors	1228		−0.9	−2.2; 0.4	0.17
Gliomas^d^	3898	1.8	0.9	0.0; 1.9	0.05
Pilocytic astrocytomas^d^	1409	1.8	−0.6	−2.0; 0.9	0.46
Other gliomas^d^	2489		1.8	0.9; 2.7	<0.01
Embryonal tumors					
Neuroblastomas	2101		−0.4	−1.4; 0.6	0.40
Retinoblastomas	742		−0.7	−2.4; 0.9	0.40
Nephroblastomas	1415		−0.1	−1.3; 1.2	0.93
Hepatoblastomas	218		1.9	−1.2; 5.1	0.23
Malignant bone and soft tissue tumors					
Malignant bone tumors	1225		−0.4	−1.7; 0.9	0.53
Osteosarcomas^d^	576	1.6	−0.9	−3.1; 1.3	0.43
Ewing tumors	556		0.7	−1.2; 2.7	0.47
Soft tissue and other extraosseous sarcomas	1654		−0.1	−1.2; 1.0	0.80
Rhabdomyosarcomas	899		0.1	−1.4; 1.6	0.88
Other soft tissue sarcomas	755		−0.5	−2.1; 1.2	0.59
Germ‐cell tumors, trophoblastic tumors, and neoplasms of gonads	836		−1.9	−3.4; −0.3	0.02
Intracranial and intraspinal GCT	229		−1.2	−4.1; 1.8	0.42
Malignant extracranial and extragonadal GCT^e^	263	1.7	−1.9	−5.1; 1.4	0.25
Malignant gonadal germ cell tumors	318		−2.6	−5.0; −0.1	0.04
Boys^e^	110		−5.4	−9.4; −1.1	0.01
Girls^e^	208		−1.1	−4.1; 2.1	0.51
All cancers	25 877		−0.1	−0.3; 0.2	0.69

AAPC: average annual percent change; CNS, Central Nervous System; GCT, Germ Cell Tumors.

Results have to be interpreted with caution.

a Number of cases.

b Φ: degree of overdispersion estimated by the ratio of the deviance of the Poisson regression model to the number of degrees of freedom.

c Average annual Percent Change and the 95% confidence interval estimated with a Poisson regression model.

d Binomial negative model.

e Less than 15 cases per year on average.

**Figure 3:**
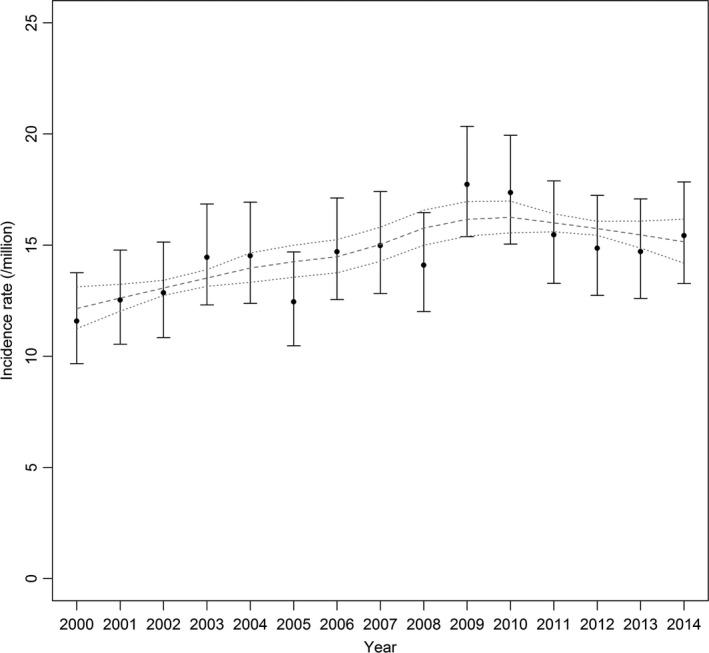
Annual incidence rate of childhood gliomas other than pilocytic astrocytomas (and 95% CI) between 2000 and 2014, and estimated loess trend (grey dashed line) with 95% confidence limits on the mean predicted values (dotted lines)

A log‐linear positive trend was significantly evidenced for gliomas (AAPC = 0.9% [0.0%; 1.9%], *P* = 0.05), in particular for gliomas other than pilocytic astrocytomas (AAPC = 1.8% [0.9%; 2.7%], *P* < 0.01). The latter increase in incidence rates was equivalent to an annual increase of three cases of gliomas other than pilocytic astrocytomas on average. Although the log‐linearity hypothesis was not significantly rejected, and no breakpoint was detected formally in joinpoint analysis, the data suggested a leveling‐off at the end of the period (Figure 3). Excluding the Occitanie region from the analyses did not change the results (not shown).

A decrease in the incidence rate of lymphomas was observed (AAPC = −1.1% [−1.9%; −0.2%], *P* = 0.01), more markedly for Burkitt lymphomas (AAPC = −2.2% [−3.8%; −0.5%], *P* = 0.01, Figure S1), and for germ cell tumors (AAPC = −1.9% [−3.4%; −0.3%], *P* = 0.02, Figure S2), with quite similar patterns in the three subgroups. The decrease in the incidence of malignant gonadal germ cell tumors appeared more marked and significant for boys (AAPC = −5.4% [−9.4%; −1.1%], *P* = 0.01) than girls (*P*_interaction_ = 0.11), with, however, only a small number of cases. No other significant interaction with gender or age was observed.

All log‐linear trends were confirmed by joinpoint analyses, and no significant time variation was evidenced for other childhood cancer groups or subgroups.

Including an intercept term for each *département* in the regression model did not change the main results.

## 4 DISCUSSION

### 4.1 Main results

A significant increase in the incidence rate of gliomas was evidenced over 2000‐2014, in particular for glioma cases other than pilocytic astrocytomas (+1.8% per year, on average), with some suggestion of a leveling‐off at the end of the study period. Some spatial heterogeneity was also observed, on the *département* scales, for pilocytic astrocytomas and other gliomas, and a large cluster, not solely responsible for the overall spatial heterogeneity, was detected in the Occitanie region. The study also suggested some spatial heterogeneity of lymphoma cases, with a large irregularly shaped cluster of NHL, and a temporal decrease in the incidence rates of Burkitt lymphomas. A decrease in the incidence rate of germ cell tumors was also suggested. Some spatial heterogeneity for neuroblastomas, on the *département* scale, and for other embryonal tumor subgroups on the LZ scale were observed but those results did not suggest a strong spatial structure and may be weak evidence.

### 4.2 Strengths and limitations

The main strengths of the study consist in the high quality incidence data, available nationwide over a long recent time period, a comprehensive description of temporal and spatial variations of childhood cancer incidence rates, with adjustment for long‐term trends of the background population and considering two geographic scales. Thanks to the high number of cases involved, analyses by diagnostic groups and subgroups were possible. We were also able to evaluate the temporal stability of the spatial results (overall heterogeneity, cluster). The fact that spatial analyses were based on count data is a limitation of the study as the results depend on the geographic scale under consideration. We focused on the LZ and *département* scale, but we cannot discard some spatial heterogeneity could exist on a different scale. Several tests were performed, which could have led to chance findings, and multiple testing was not formally adjusted for. However, the statistical significance levels of the main results were small and they were consistent with respect to sensitivity analyses.

### 4.3 Classification of childhood cancers

The study benefited from population estimates from national censuses and exhaustive data on childhood cancer over a long time period from the national registries. Analyses were conducted for the main diagnostic groups, as defined by the International Classification of Childhood Cancer, Third edition, and their main subgroups. In order to avoid misclassification bias for CNS tumors, a large group of gliomas was considered as a whole and then split into pilocytic astrocytoma and other glioma subgroups. The extensive use of magnetic resonance imaging and immunohistochemical staining has improved the sensitivity and accuracy of histological diagnosis of gliomas and recent progress in molecular methods has enabled enhanced characterization of gliomas. However, those diagnostic techniques may not be widely available or widely used. Diagnosis of glioma may be tricky^14^ and glioma classification by histological subtype may depend on the diagnostic methods available and the pathologist's experience. In some situations, medical records are reviewed by several experts and disagreements on diagnosis are not unusual, mainly with regard to glioma grade and subtype.^15^ Distinguishing between malignant and not malignant CNS tumors is not easy either. Given these points, spatial and/or temporal variations may be observed and misinterpreted as genuine variations. Considering pilocytic astrocytomas separately from other gliomas should reduce the bias associated with classification errors, as pilocytic astrocytoma is a relatively well‐characterized glioma.

### 4.4 Temporal variations of childhood cancers

In the main analyses, temporal variations of childhood cancer over the 2000‐2014 period were described with a log‐linear Poisson regression model. The Joinpoint regression method was also used to test for a piecewise linear variation in the incidence rate. The latter method is well adapted to detect marked changes in the linear slope but it may be not powerful enough in cases of gradual variations in the annual incidence rate. When a nonlinear trend was graphically suggested, we thus considered a smoothed general additive model.

All things considered, no sustained increase in the incidence rate of childhood cancer in France between 2000 and 2014 was observed. An increase was observed for gliomas other than pilocytic astrocytomas, but the data suggested some stability in more recent years. A decreasing trend was evidenced for Burkitt lymphomas and gonadal germ cell tumors. A decreasing trend was also suggested for other germ cell tumors subgroups. The number of sources of registration per case of Burkitt lymphoma or germ cell tumor (3.0 and 2.9 on average, respectively) did not support case under‐ascertainment in the most recent years. However, a few cases may have been missed by the registry if they were diagnosed and treated in nonpediatric hospital departments that are not routinely covered by active searching. Additional years of registration are needed to confirm the observed decreasing trends.

The main results of the studies published since 2000 are presented as supporting information with a separate table for each of the 10 main groups of childhood cancer (Tables S1‐S10). For childhood leukemia (Table S1), for which the literature is particularly abundant, we have updated the summary table provided by Maule *et al*.^16^ by reporting the publications since 2005. Several studies have evaluated the temporal trends in childhood cancer incidence since the 1970s‐1980s in various countries and, in line with the recent update of the IICC study,^17^ they showed an increase in the incidence rates for several types of childhood cancer. Improvements in diagnostic methods and cancer registration have certainly played a role in the reported increases, but are less likely to have done so over the most recent periods.

Thus, we discussed the studies that were conducted over recent time periods and, among the remaining studies, those that considered nonlinear or piecewise linear time variations. The results were relatively concordant for CNS tumors, retinoblastomas, renal tumors, and bone tumors with no significant variations reported (Tables S3, S5, S6, S8), while the results were more heterogeneous for other cancer groups (Tables S1, S2, S4, S7, S9, S10).

In particular, the decreasing trends we observed over 2000‐2014 were not found consistently by other recent studies. At the present time, no clear conclusion can be drawn for lymphomas (Table S2): a positive significant trend was observed in nine studies,^18^, ^19^, ^20^, ^21^, ^22^, ^23^, ^24^, ^25^, ^26^ with different results by subgroup of lymphoma, and with a leveling off in the most recent years for three of those studies^20^, ^22^, ^26^; four studies generated nonsignificant results^27^, ^28^, ^29^, ^30^ (for White non‐Hispanics in Gittleman *et al*.^28^); and only one study, conducted in Mexico, evidenced a significant decreasing trend, over 1996‐2010.^31^

The results for germ cell tumors over recent periods were also quite heterogeneous (Table S10): An increase was suggested in Australia^18^ and Taiwan,^19^ but not in the USA,^21^ Germany,^32^, ^33^ Great Britain^20^ or Canada.^29^ Results for the USA are not clear‐cut but no increase was found overall.^21^, ^23^, ^24^

As previously noted by Maule et al^16^ who summarized papers published before 2005, the results from the recent literature are heterogeneous for leukemia (Table S1): Since 2005, ten studies found an increase, which persisted beyond 2000,^18^, ^19^, ^20^, ^24^, ^25^, ^26^, ^28^, ^30^, ^34^, ^35^ while eight other studies did not find any significant variation either over the whole study period^21^, ^23^, ^36^ or over the most recent years.^16^, ^22^, ^29^, ^37^, ^38^

The persistence of an increase has also been discussed for brain tumors, the most frequent cancer in children after leukemia. Overall, the literature does not support a positive trend over the most recent years (Table S3). Only three studies of sixteen actually found a significant increase over a quite recent period.^16^, ^19^, ^28^

### 4.5 Spatial variations of childhood cancers

For an in‐depth description of the country‐wide spatial variations of childhood cancer in France, we considered two geographic scales: the 95 *départements*, and the 1916 living zones (LZ). Because of its size, the *département* scale is appropriate to describe the overall spatial structure of childhood cancer but not to detect spatial heterogeneity on a finer scale or detect small localized clusters. The LZ scale is of particular value for the description of childhood environments because, as the name suggests, the living zone constitutes an area in which people live and work. However, that scale is associated with small numbers of cases by unit, which may be a limitation for clustering analyses and the detection of clusters. In situations where a large number of geographic units are considered, and few of them contribute two or more cases, as is the case in this study, interpretation of the results may be problematic. For clustering analyses, we simultaneously considered the results from three complementary tests (Potthoff and Whittinghill's, Moran's and Rogerson's tests), and both geographic scales, in order to highlight the main results and avoid overinterpretation of false‐positive results.

In addition to clustering methods that aim to detect global spatial heterogeneity, several methods were developed to detect localized excesses of cases (clusters). Easy to implement with the publicly available software, Kulldorff's spatial scan method (SaTScan method) is the most well‐known method and has been widely used. The FleXScan method is more computer intensive, particularly with a large number of geographic units, but has the advantage of being able to detect arbitrarily shaped clusters. In a previous study,^37^ we evaluated the statistical power of both methods and four other cluster detection methods in several realistic situations of childhood cancer clusters on the LZ scale. All methods performed equally well in detecting large compact clusters but SaTScan and FleXScan were the best methods for the detection of small compact or linear clusters, and U‐shaped clusters, respectively. However, in several alternative situations, we noted that at most half of the LZ included in the true cluster were well identified and several LZ were wrongly detected. False‐negatives and false‐positives are less likely on the *département* scale. It is noteworthy, besides, that all the cluster detection methods failed to detect small clusters with low or moderate relative risks. Hence, the presence of small localized excesses of some childhood cancers in France cannot be ruled out.

CNS tumor cases, in particular glioma cases, tended to be overdispersed on the LZ scale and more markedly on the *département* scale. At first sight, the overdispersion parameter estimate seemed quite elevated on the *département* scale (0.67) relative to its value on the LZ scale (0.10), and relative to the overdispersion index reported in previously published studies on childhood cancer (mostly lower than 0.05, Table S11). However, the variance of the overdispersion parameter estimate is inversely related to the total number of geographic units under consideration and most of the studies that used Potthoff and Whittinghill's test were conducted on a large territory with a large number of areas (10 444 wards in UK,^39^ 36 600 municipalities in France,^40^ 12 262 municipalities in Germany^41^). If we consider standardized estimates, the overdispersion parameters are of the same order of magnitude (in the present study, 4.6 and 3.0 for gliomas on the *département* and LZ scales, respectively; eg 5.4 and 3.3 for all cancer cases and leukemia cases, respectively, on the ward scale in UK^39^). A cluster of glioma cases, mostly due to an excess of gliomas other than pilocytic astrocytomas, was detected both by SaTScan and FleXScan in the southern region of France. The location and shape of the cluster area cannot be determined precisely on the LZ scale. The methods were in closer agreement on the *département* scale, with an intersection of nine *départements* with 303 observed cases and 206.5 expected cases. When the glioma group was restricted to cases with a histologically confirmed diagnosis, the systematic search did not detect any cluster area. The latter result suggests that the excess of glioma cases could be due to an excess of cases with no histological confirmation of their diagnosis, which may be related to spatial differences in registration or diagnostic practices.

Overdispersion was also evidenced for non‐Hodgkin lymphomas on the *département* scale but the results were not stable by period and therefore not in favor of strong spatial heterogeneity. A large irregularly shaped cluster of non‐Hodgkin lymphomas was also detected in a region located in the Center‐east of France, with a relative risk of about 1.5 for Burkitt lymphomas and other non‐Hodgkin lymphomas. At the present time, we do not have any explanation for the presence of that cluster.

Spatial variations of childhood leukemia have been widely investigated and for a long time the disease has been said to have a general, but weak, tendency toward spatial clustering.^3^, ^5^, ^42^ Due to the rarity of childhood cancer, several studies have investigated spatial variations combining several years of registrations, sometimes starting from the 1970s to 1980s.^39^, ^43^, ^44^, ^45^ As we discussed for temporal variations, progress in diagnosis and registration may be responsible for some spatial differences in incidence rates over those periods. No overall spatial heterogeneity (clustering) was evidenced consistently in two U.S. states,^46^, ^47^ Germany,^41^ Spain,^48^ Hungary,^49^ or Switzerland^1^ (Table S11). Spatial heterogeneity was suggested in France on the *commune* scale (about 36 600 units), with a small effect restricted to the period 1990‐1994,^40^ but it was not observed on the LZ scale for the period 1990‐2006^50^ and not observed either for 2000‐2014 in the present study.

Spatial variations of other childhood cancers have been markedly less investigated and the main epidemiological information comes from Great Britain, with a large study based on the British national registry of childhood tumors.^39^ However, the study covered a long time period (1969‐1993) during which case registration procedures and diagnostic methods improved.^20^ So the results have to be interpreted with caution. Spatial variations of childhood cancer were also investigated in the Manchester area, with data from the Manchester childhood tumor registry. Except for CNS tumors,^51^ for which no spatial heterogeneity was observed, most of the analyses were based on small numbers of cases. More recently, no strong spatial heterogeneity of lymphomas or childhood CNS tumors was evidenced in five regions of Spain.^48^

Several studies have also identified some localized excesses of childhood cancer cases (Table S11), without clinical implication or etiological hypothesis.

## 5 CONCLUSION

With more than 25 000 cases registered nationwide, the present study provides substantial information on the spatial and temporal variations of the main types of childhood cancer, leukemias, lymphomas and brain tumors, and other solid tumors that have been less investigated.

The incidence rates in France tended to vary between 2000 and 2014 for some particular groups of childhood cancer, with an increase for gliomas, and a decreasing trend for lymphomas and germ cell tumors, but, overall, the results do not support a sustained increase since 2000.

Several types of childhood cancer displayed some small spatial heterogeneity, in particular on the LZ scale. CNS tumors, in particular gliomas, were overdispersed on both geographic scales and a large cluster was detected. Non‐Hodgkin lymphomas also exhibited some spatial heterogeneity on the *département* scale and a widespread irregularly shaped cluster was detected. The finding of persistent localized excesses of cases may reflect the presence of localized risk factors but, as yet, we have no strong explanation for the large clusters of gliomas and non‐Hodgkin lymphomas, and spatial differences in case ascertainment cannot be ruled out. Interestingly, no spatial heterogeneity of childhood leukemia, or other solid tumor groups, was evidenced.

## CONFLICT OF INTEREST

None declared.
