# Supplementary material for: Spatial and temporal variations of childhood cancers: Literature review and contribution of the French national registry
Source: Cancer Med. 2018 Sep 19;7(10):5299–314. doi: 10.1002/cam4.1774 (PMC6198217; doi:10.1002/cam4.1774)
Supplement: Supplementary file 1 [file CAM4-7-5299-s001.docx]

Supplementary material

Spatial and temporal variations of childhood cancers: the French national registry's contribution to worldwide knowledge

**Contents**

**Supplementary tables 1-10** – Time variation in the incidence of childhood cancer - main results of papers published since 2000, by diagnostic group:

- Supplementary table 1 (page 2): leukemia
- Supplementary table 2 (page 5): lymphomas
- Supplementary table 3 (page 7): brain tumors
- Supplementary table 4 (page 10): peripheral nervous cell tumors
- Supplementary table 5 (page 11): retinoblastomas
- Supplementary table 6 (page 12): renal tumors
- Supplementary table 7 (page 13): hepatic tumors
- Supplementary table 8 (page 14): bone tumors
- Supplementary table 9 (page 15): soft tissue and other extraosseous sarcomas
- Supplementary table 10 (page 17): germ cell tumors

**Supplementary table 11** (page 19) Spatial variation in the incidence of childhood cancer - main results of the papers published since 2000

**Supplementary table 12** (page 23) Distribution of the person-years at risk by year of age from 2000 to 2014, mainland France (column percentages)

**Supplementary figure 1** (page 24) Annual incidence rate of childhood Burkitt lymphomas (and 95% CI) between 2000 and 2014 and estimated log-linear trend (dashed line)

**Supplementary figure 2** (page 25) Annual incidence rate (and 95% CI) of childhood malignant germ-cell tumors between 2000 and 2014 and estimated log-linear trend (dashed line)

**References** (sorted by author and year), page 27

**Supplementary table 1** Time variation in the incidence of childhood leukemia - main results of papers published since 2005 (not referred to in [Maule, et al. 2006](#_ENREF_36))^(a)^

| **Reference** | **Country** | **Source of data**^(b)^ | **Period**^(c)^ | **N**^(d)(e)^ | | | **Main results (AAPC if not otherwise specified)**^(e)^ |
| --- | --- | --- | --- | --- | --- | --- | --- |
| Studies with results on recent temporal trend^(a)^ | | | | | | | |
| [Agha, et al. 2006](#_ENREF_1)^(f)^ | Canada, Ontario | POGO | 1986-2001 | 1,642 | AL |  | 0.2% [0.8;1.3] |
| [Maule, et al. 2006](#_ENREF_36) ^(f)^ | Italy, Piedmont | CCRP reg. | 1975-2001 | 688 | ALL |  | - Studies published over 1966-2005 in western countries   “The overall pattern from these studies is that the incidence of all leukemias, and especially acute lymphoblastic leukemia (ALL), has been either increasing or stable over the last few decades. Thus, there are differences in the time trends between and sometimes within geographical areas. […] It is also noteworthy that the increases in incidence were greatest in the earlier years of observation, while incidence has been more stable since the 1980s”   - Maule et al. study results   ALL “increase until 1997 then decrease” ANLL (145 cas) NS |
| [Linabery and Ross 2008](#_ENREF_34)^(f)^ | USA | SEER, 13 reg. | 1992-2004 | 6,129 | AL | 0-19 y.o. | 0.7% [-0.1;1.5] AL  0.8% [-0.4;1.9] ALL  1.0% [-0.4;2.4] AML |
| [Spix, et al. 2008](#_ENREF_62) | Germany | GCCR reg. | 1987-2004  1991-2004 | 6,958  1,197 | AL  AL | West  East | 0.6% [0.2;1.1] AL; 0.7% [0.2;1.2] ALL; 0.3% [-0.8;1.4] ANLL  2.1% [0.7;3.5] AL; 2.1% [0.6;3.7] ALL; 1.3% [-2.2;5.0] ANLL |
| [Baade, et al. 2010](#_ENREF_4) ^(f)^ | Australia | APCR reg. | 1983-2006 | 4,591 | AL |  | 0.9% [0.3;1.5] |
| [Peris-Bonet, et al. 2010](#_ENREF_49)^(f)^ | Spain | 11 regional reg. | 1983-2002 | 2,478 | HM |  | 1983-2002: 1.6% [0.8;2.3];  1983-1986: 5.0% [2.5;8.3]; 1996-2002: -0.3% [-2.2;1.4] |
| [Kroll, et al. 2012](#_ENREF_31) ^(f)^ | Great Britain | NRCT reg. | 1966-2005 | 17,850 | AL |  | 0.7% [0.6;0.9]; step in 1971, 1990, 2002 |
| [Mitra, et al. 2012](#_ENREF_44)^(f)^ | Canada | CCR reg. | 1992-2006 | 4,287 | AL |  | 2.4% [0.0;4.9] 1992-1999; -4.4% [-20.1;14.2] 1999-2002  3.0% [-2.6;9.0] 2002-2006 |
| [Goujon-Bellec, et al. 2013](#_ENREF_18)^(f)^ | France | NRCH | 1990-2007 | 6,686 | ALL |  | 0.5% [0.0;1.0]  2.2% [1.1;3.2] for 7-14 y.o. pre-B cell ALL (leveling off since 2001) |
| [Petridou, et al. 2013](#_ENREF_50) | Greece, Bulgarian  Turkey  Russia | NARECHEM reg.  BNCR reg.  Antalya reg.  Izmir reg.  Moscow reg. | 1996-2010 1996-2009  1998-2008 1996-2007 2000-2010 | 1,215  675  403 323 425 | AL  AL  AL  AL  AL |  | 1.7% [0.4;3.0] p=0.01 3.5% [1.6;5.5] p=<0.001 0.9% [-8.7;11.5] p=0.86 2.7% [-0.1;5.6] p=0.06 2.7% [-0.3;5.9] p=0.08 |
| [Hung, et al. 2014a](#_ENREF_20) ^(f)^ | Taiwan | TCR reg. | 1995-2009 | 3,520 | AL | 0-19 y.o. | 2.3% [1.7;3.0]; Girls: 1.6% [0.4;2.8]; trend unclear for Boys |
| [Siegel, et al. 2014](#_ENREF_61) | USA | NPCR(CDC), SEER | 2001-2009 | 31,824 | AL | 0-19 y.o. | 0.5% [-0.3;1.3] |
| [Svendsen, et al. 2007](#_ENREF_66) ^(f)^ | Denmark Finland Norway Sweden | National reg. | 1976-2002 | 4,403 | ALL |  | 3.3% [1.5;5.2] before 1983; 0.2% [-0.3;0.8] after 1983 2.5% [1.4;3.6] before 1980; 0.1% [-0.5;0.6] after 1980 (birth cohorts) |
| [Ward, et al. 2014](#_ENREF_69)^(f)^ | USA | SEER, 9 reg. | 1975-2010 | n.a. | AL | 0-19 y.o. | ALL: 0.7% significant result (Fig.2) AML: 1.1% significant result (Fig 2) |
| [Demanelis, et al. 2015](#_ENREF_13)^(f)^ | Thailand  USA | SCR3 reg.  SEER, 9 reg. | 1990-2011 | 316  6,738 | AL | 0-19 y.o. | SCR reg.: AL: 1.7% p=0.03; ALL: 1.8% p=0.03; AML 4.2% p=0.04  SEER: AL: 0.8% p<0.001; ALL: 0.9% p<0.001; AML 0.3% p=0.54 |
| [Gittleman, et al. 2015](#_ENREF_17)^(f)^ | USA | CBTRUS reg.  US Cancer Statistics | 2000-2010 | n.a.  n.a. | ALL  AML |  | 1.0% [0.5;1.5] 0.5% [-0.4;1.5] |
| [Barrington-Trimis, et al. 2017](#_ENREF_6)^(f)^ | USA | SEER, 13 reg. | 1992-2013 | n.a. | AL | 0-19 y.o. | Hispanic White children 1.1 [0.6;1.6]  NS for non-Hispanic White, Black or Asian children |
| [Isaevska, et al. 2017](#_ENREF_22) ^(f)^ | Italy (Piedmont) | CCRP reg. | 1976-2011  2000-2011 | 1,168  931  378  306 | AL  ALL  AL  ALL |  | 0.6% [0.0;1.2]  -0.6% [-5.4;4.4]  0.9% [0.3;1.4]  -0.2% [-4.7;4.5] |
| Other studies ^(a)^ | | | | | | | |
| [Steliarova-Foucher, et al. 2004](#_ENREF_64) | Europe | ACCIS, 63 reg. | 1970-1999 | n.a.  n.a. | AL  ALL |  | 0.7% p<0.0001 1.4% p<0.0001 |
| [Dalmasso, et al. 2005](#_ENREF_12) | Italy (NW, Piedmont) | CCRP reg. | 1967-2001 | 1,121 | AL |  | 1.0% [0.4;1.6] AL  1.2% [0.2;2.3] ALL (1-4 y.o. 1.8% [0.3;3.3])  1.7% [-0.6;4.1] ANLL (10.14 y.o.: 4.4% [0.0;9.0]) |
| [Kaatsch, et al. 2006](#_ENREF_28) | Europe | ACCIS, 33 reg. | 1978-1997 | 26,690 | AL |  | 0.6% p<0.0001 AL  0.8% p<0.0001 ALL |
| [Kamsa-ard, et al. 2006](#_ENREF_29) | Thailand | KKCR reg. | 1985-2002 | 277 | AL |  | 2.4% [-0.5;5.3] boys  4.1% [1.1;7.2] girls |
| [Kroll, et al. 2006](#_ENREF_32) | England | NRCT reg. | 1974-2000 | 9,452 | ALL |  | 0.7% p<0.05 ALL 1.4% p<0.05 pre B cell ALL over 1980-1996 |
| [Shah and Coleman 2007](#_ENREF_60) | England  Wales | ONS | 1971-2000 | n.a. | AL |  | 1.5% [1.5;1.5] quinquennial change AL  2.4% [1.0;3.8] for < 1 y.o.; 2.8% [1.7;3.8] for 1-4 y.o. |
| [Kaatsch and Mergenthaler 2008](#_ENREF_27) | Germany, EUROPE | GCCR reg.  ACCIS | 1987-2004 | 8,388  26,690 | AL  AL |  | AL: GCCR: 0.6% p<0.01; ACCIS: 0.6% p<0.001 ALL: GCCR: 0.7% p<0.01; ACCIS: 0.8% p<0.001 AML: GCCR: 0.3% NS; ACCIS: 0.5% NS |
| [Bao, et al. 2010](#_ENREF_5) | Shanghai | SCR2 reg. | 1973-2005 | 1,107 | AL | (ICD-9) | ALL Boys: -0.9% [-4.5;2.8] Girls: -2.3% [-6.6;2.3]  AML Boys: -8.6% [-15.2;-1.6] Girls: -9.5% [-16.2;-2.3] |
| [Kulkarni, et al. 2011](#_ENREF_33) | Canada | ACR reg. | 1982-2004 | 525 117 | ALL AML | 0-19 y.o. | “With relatively small counts for the individual diagnoses, no clear trends emerged” |
| [Linet, et al. 2016](#_ENREF_35) | International (cancer in 5 continents) | IARC, SEER | 1978-2007 | n.a. | HM | 0-19 y.o. | "Based on temporal trends during 1978–2007 in 24 populations, lymphoid leukemia and myeloid leukemia incidence rates generally have not changed greatly" |

^(a)^ The first part of the table includes the results from 1) studies based on a recent time period (starting in 1990 or after), and 2) studies based on a time period starting before 1990, with consideration of temporal variations in the most recent years (mainly with nonlinear modeling). The second part of the table includes the studies that considered a log-linear temporal trend on a long time period starting before 1990. Improvements in diagnostic methods and cancer registration have certainly played a role in the increases reported in the latter studies (cf discussion section in the manuscript). ^(b)^ Reg.: registry; ACCIS: Automated Cancer Information System; ACR: Alberta Cancer Registry; APCR: Australian Paediatric Cancer Registry; BNCR: Bulgarian National Cancer Registry; CCR: Canadian Cancer Registry; CCRP: Childhood Cancer Registry of Piedmont; CDC: Centers for Disease Control and Prevention; ECR: Eindhoven Cancer Registry; GCCR: German Childhood Cancer Registry; IARC: International Agency for Research on Cancer; KKCR: Khon Kaen Cancer registry; MCTR: Manchester Children's Tumour Registry; NARECHEM: Nationwide Registry for Childhood Hematological Malignancies; NOR: National Oncology Registry; NPCR: National Program of Cancer Registries; NRCT: national Registry of Childhood Tumors; NRYPMDR:: Northern Region Young Persons’ Malignant Disease Registry; NSPHO: Nordic Society of Paediatric Haematology and Oncology; ONS: Office for National Statistics; POGO: Pediatric Oncology Group of Ontario; RNHE: French National Registry of Childhood hematological malignancies; SCR^1^: Swedish Cancer Registry; SCR^2^: Shanghai Cancer Registry; SCR^3^: Songkhla cancer registry; SEER: Surveillance; Epidemiology, and Results Program; TCR: Taiwan Cancer Registry; YSRCCYP: Yorkshire Specialist Register of Cancer in Children and Young People. ^(c)^ period considered for time-trend; ^(d)^ Number of cases (N) and diagnostic group (0-14 y.o. if not otherwise specified); n.a.: not available; ^(e)^ AL: acute leukemia; ALL: acute lymphoid leukemia; AML: acute myeloid leukemia; HM: hematologic malignancy; Pre-B cell ALL: precursor B-cell acute lymphoblastic leukemia; ^(f)^ nonlinear variation was considered.

AAPC: Average Annual Percent Change; NS: Not Significant according to the authors.

**Supplementary table 2** Time variation in the incidence of childhood lymphomas - main results of the papers published since 2000^(a)^

| **Reference** | **Country** | **Source of data**^(b)^ | **Period**^(c)^ | **N**^(d)(e)^ | | | **Main results (AAPC if not otherwise specified)**^(e)^ |
| --- | --- | --- | --- | --- | --- | --- | --- |
| Studies with results on recent temporal trend^(a)^ | | | | | | | |
| [Clavel, et al. 2004](#_ENREF_9) | France | RNHE reg. | 1990-1999 | 1,037 | NHL |  | NS |
| [Agha, et al. 2006](#_ENREF_1)^(f)^ | Canada | POGO | 1986-2001 | 498 | Lymph |  | 1.9% [0.0;3.9] |
| [Linabery and Ross 2008](#_ENREF_34) ^(f)^ | USA | SEER, 13 reg. | 1992-2004 | 1,611  1,472 | HL  NHL | 0-19 y.o. | -0.8% [-2.4;0.7]  1.4% [-0.1;3.0] |
| [Spix, et al. 2008](#_ENREF_62) | Germany | GCCR reg. West  East | 1987-2004  1991-2004 | 2,947 564 | Lymph |  | 0.9% [0.2;1.6]; HL: 1.6% [-4.5;8.0]; NHL: 0.1% [-0.9;1.2]  -0.9% [-3.0;1.2]; HL: -2.0% [-5.1;1.1]; NHL: 0.2% [-2.8;3.4] |
| [Baade, et al. 2010](#_ENREF_4)^(f)^ | Australia | APCR reg. | 1983-2006 | 1,374 | Lymph |  | 0.7% [0.0;1.3] |
| [Peris-Bonet, et al. 2010](#_ENREF_49)^(f)^ | Spain | 11 regional reg. | 1983-2002 | 2,478 | HM |  | 1983-2002: 1.6% [0.8;2.3];  1983-1986: 5.0% [2.5;8.3]; 1996-2002: -0.3% [-2.2;1.4] |
| [Kroll, et al. 2012](#_ENREF_31) ^(f)^ | Great Britain | NRCT reg. | 1966-2005 | 5,579 | Lymph |  | 0.8% [0.6;1.1] step in 1976 and 1997 |
| [Mitra, et al. 2012](#_ENREF_44) ^(f)^ | Canada | CCR reg. | 1992-2006 | 1,480 | Lymph |  | 0.0% [−1.4 to 1.4] |
| [Petridou, et al. 2013](#_ENREF_50) | Greece, Bulgarian  Turkey  Russia | NARECHEM reg.  BNCR  Antalya reg.  Izmir reg.  Moscow reg. | 1996-2010 1996-2009  1998-2008 1996-2007 2000-2010 | 422 292 158 121 190 | Lymph |  | -0.5% [-2.7;1.7] p=0.64 -2.1% [-4.8;0.8] p=0.15  2.3% [-12.6;19.8] p=0.78  3.7% [-0.5;8.1] p=0.08 -3.0% [-7.6;1.8] p=0.22 |
| [Hung, et al. 2014a](#_ENREF_20) ^(f)^ | Taiwan | TCR reg. | 1995-2009 | 1,470 | Lymph | 0-19 y.o. | 2.2% [0.7, 3.7]; Boys: 1.3% [-0.4, 3.1]; Girls: 4.0% [1.1, 7.0] |
| [Siegel, et al. 2014](#_ENREF_61) | USA | NPCR(CDC), SEER (47 reg.) | 2001-2009 | 17,445 8,780 1,712 6,047 | All  HL  Burkitt  Other | 0-19 y.o. | 0.5% [-0.2;1.3]; Boys: 0.8% [-0.3;1.9]; Girls: 0.1% [-0.9;1.2] 0.5% [-0.3;1.3]; Boys: 0.5% [-0.7;1.7]; Girls: 0.5% [-1.1;1.2] -0.1% [-2.7;2.7]; Boys: 0.0% [-2.9;2.9]; Girls: 0.1% [-4.7;5.1] 0.7% [-0.5;1.8]; Boys: 1.3% [0.0;2.5]; Girls: -0.4% [-1.3;0.6] |
| [Ward, et al. 2014](#_ENREF_69) ^(f)^ | USA | SEER, 9 reg. | 1975-2010 |  | Lymph | 0-19 y.o. | NHL: 1.1% HL: -0.7% |
| [Gittleman, et al. 2015](#_ENREF_17)^(f)^ | USA | CBTRUS reg.  US Cancer Statistics | 2000-2010 | n.a.  n.a. | HL  NHL |  | -0.1% [-1.5;1.3]  0.6% [0.1;1.0] (white non-hispanics: 0.3% [-0.2;0.9]) |
| [Rendon-Macias, et al. 2015](#_ENREF_53) | Mexico | MCCR reg. | 1996-2010 | 328 | Lymph |  | -3.5% p<0.05 |
| [Isaevska, et al. 2017](#_ENREF_22) ^(f)^ | Italy (Piedmont) | CCRP reg. | 1976-2011  2000-2011 | 449  151 | Lymph |  | 1976-2006: 1.7% [0.6;2.7] ; 2007-2011 : -12.2% [-33.2;15.4]  2000-2011 : less than 5 cases/year |
| Other studies^(a)^ | | | | | | | |
| [Cotterill, et al. 2000](#_ENREF_11) | UK | NRYPMDR reg. | 1968-1995 | 1,896 | HM |  | 3.6% [-0.4;7.6%] per decade (figure 2) |
| [McNally, et al. 2001](#_ENREF_42) | Engl, Manchester | MCTR reg. | 1954-1998 | 173 195 | HL  NHL |  | 1.2% [0.1;2.4]  0.2% [-0.9;1.3] |
| [Dreifaldt, et al. 2004](#_ENREF_16) | Sweden | SCR^1^ reg. | 1960-1998 | 947 | Lymph |  | 1.9% [1.2;2.6] |
| [Steliarova-Foucher, et al. 2004](#_ENREF_64) | Europe | ACCIS, 63 reg. | 1970-1999 | n.a.  n.a. | Lymph  HL |  | 1.3% p<0.0001 1.5% p<0.0001 |
| [Dalmasso, et al. 2005](#_ENREF_12) | Italy (NW, Piedmont) | CCRP reg. | 1967-2001 | 395  156  150 | Lymph  HL  NHL |  | 1.4% [-0.3;3.0]; 10-14 y.o.: 3.6% [2.0;5.2] 1.4% [-0.3;3.0]; 10-14 y.o.: 2.6% [0.0;4.8] 0.8% [-2.4;0.9] |
| [Clavel, et al. 2006](#_ENREF_10) | Europe | ACCIS, 33 reg. | 1978-1997 | 3,628 | HL |  | 1.0% [0.3;1.6] |
| [Izarzugaza, et al. 2006](#_ENREF_23) | Europe | ACCIS, 33 reg. | 1978-1997 | 5,343 | NHL |  | 0.9% p=0.002 |
| [Kaatsch, et al. 2006](#_ENREF_28) | Europe | ACCIS, 33 reg. | 1978-1997 | 8,971 | Lymph |  | 0.9% p<0.0001 |
| [Bao, et al. 2010](#_ENREF_5) | Shanghai | SCR2 reg. | 1973-2005 | 58  320 | HL  NHL |  | -11.7% [-22.4;0.5] Boys; -5.6% [-18.8;9.6] Girls  2.1% [-1.8;6.2] Boys; 9.3% [-0.1;19.6] Girls |
| [Srina, et al. 2010](#_ENREF_63) | Thailand | KKPR reg. | 1985-2008 | 72 | Lymph |  | -0.3% [-6.3;5.8] |
| [Kulkarni, et al. 2011](#_ENREF_33) | Canada | ACR reg. | 1982-2004 | 257 111 | HL  NHL | 0-19 y.o. | "With relatively small counts for the individual diagnoses, no clear trends emerged" |
| [Hjalgrim, et al. 2016](#_ENREF_19) | Nordic countries | National reg. | 1978-2010 | 748 | HL |  | Interaction with age Decreasing trend for younger children and increase for older children |
| [Linet, et al. 2016](#_ENREF_35) | International (cancer in 5 continents) | IARC, SEER | 1978-2007 | n.a. | HM | 0-19 y.o. | "Based on temporal trends during 1978–2007 in 24 populations, […] differences in rates for non-Hodgkin and for Hodgkin lymphoma have diminished in some regions." |

^(a)^ The first part of the table includes the results from 1) studies based on a recent time period (starting in 1990 or after), and 2) studies based on a time period starting before 1990, with consideration of temporal variations in the most recent years (mainly with nonlinear modeling). The second part of the table includes the studies that considered a log-linear temporal trend on a long time period starting before 1990. Improvements in diagnostic methods and cancer registration have certainly played a role in the increases reported in the latter studies (cf discussion section in the manuscript). ^(b)^ Reg.: registry; ACCIS: Automated Cancer Information System; ACR: Alberta Cancer Registry; APCR: Australian Paediatric Cancer Registry; BNCR: Bulgarian National Cancer Registry; CCR: Canadian Cancer Registry; CCRP: Childhood Cancer Registry of Piedmont; CDC: Centers for Disease Control and Prevention; ECR: Eindhoven Cancer Registry; GCCR: German Childhood Cancer Registry; IARC: International Agency for Research on Cancer; KKCR: Khon Kaen Cancer registry; MCCR: Mexican Childhood Cancer Registry; MCTR: Manchester Children's Tumour Registry; NARECHEM: Nationwide Registry for Childhood Hematological Malignancies; NPCR: National Program of Cancer Registries; NRCT: national Registry of Childhood Tumors; NRYPMDR:: Northern Region Young Persons’ Malignant Disease Registry; POGO: Pediatric Oncology Group of Ontario; RNHE: French National Registry of Childhood hematological malignancies; SCR^1^: Swedish Cancer Registry; SCR^2^: Shanghai Cancer Registry; SEER: Surveillance, Epidemiology, and Results Program; TCR: Taiwan Cancer Registry. ^(c)^ period considered for time-trend; ^(d)^ Number of cases (N) and diagnostic group (0-14 y.o. if not otherwise specified); n.a.: not available; ^(e)^ HL: Hodgkin lymphoma; HM: hematological malignancy; Lymph: lymphoma; NHL: non-Hodgkin lymphoma; Other spec.: other specified lymphoma; ^(f)^ nonlinear variation was considered.

AAPC: Average Annual Percent Change; NS: Not Significant according to the authors.

**Supplementary table 3** Time variation in the incidence of childhood brain tumors - main results of the papers published since 2000

| **Reference** | **Country** | **Source of data**^(a)^ | **Period**^(b)^ |  | **N**^(c)(d)^ |  | **Main results (AAPC if not otherwise specified)** |
| --- | --- | --- | --- | --- | --- | --- | --- |
| Studies with results on recent temporal trend^(a)^ | | | | | | | |
| [Agha, et al. 2006](#_ENREF_1) ^(e)^ | Canada, Ontario | POGO | 1986-2001 | 1,203 | CNS |  | -1.4% [-3.2%;0.4] (leveling off since 1990) |
| [Maule, et al. 2006](#_ENREF_36)^(e)^ | Italy, Piedmont | CCRP reg. | 1975-2001 | 753 | CNS |  | 2.3% [1.6;3.0] (Poisson model) |
| [Linabery and Ross 2008](#_ENREF_34)^(e)^ | USA | SEER, 13 reg. | 1992-2004 | 4,004  308  1,920  971  664  141 | CNS  IIIa.  IIIb.  IIIc.  IIId.  IIIe+IIIf | 0-19 y.o. | -0.1% [-1.1;1.0]  -0.5% [-4.1;3.3] -2.9% [-5.2;0.1] 1992-1999; +7.8% [-9.1;27.9] 1999-2002; -8.3% [-23.1;9.4] 2002-2004 -1.2% [-3.1;0.7]  1.5% [-0.9;4.0]  5.4% [0.8;10.3] |
| [Baade, et al. 2010](#_ENREF_4) ^(e)^ | Australia | APCR reg. | 1983-2006 | 3,158 | CNS |  | 1.7% [0.6;2.8] 1983-1998; -1.8% [-4.5;1.0] 1998-2006 |
| [Peris-Bonet, et al. 2010](#_ENREF_49) ^(e)^ | Spain | 11 cancer reg. | 1983-2002 | 1,291  n.a. | CNS  Malign. |  | 2.2% [1.2;3.2] 1983-2002 (4.3% [1.7;8.5] 1983-1985; -0.1% [-6.1;3.0] 2001-2002)  1.5% [0.4;2.5] 1983-2002 (3.6% [1.3;6.9] 1983-2001; -2.6% [-13.0;1.6] 2001-2002) |
| [Schmidt, et al. 2011](#_ENREF_55) ^(e)^ | Nordic countries (Denmark, Norway, Finland, Sweden) | Cancer reg. NSPHO database | 1985-2006 | 3,983 405 1,710 692 332 541 303 | CNS IIIa IIIb IIIc IIId IIIe IIIf |  | 0.03% NS -0.1% [-1.4;1.3] -1.3% [-1.9;-0.2]   1.0% [0.0;1.9] -0.6% [-2.6;1.4]  4.2% [-0.5;9.0]   0.2% [-2.1;2.6] |
| [Kroll, et al. 2012](#_ENREF_31)^(e)^ | Great Britain | NRCT | 1966-2005 | 13,622 | Brain | (III+Xa) | 1.3% [1.2;1.5] (leveling off since 1992) |
| [Mitra, et al. 2012](#_ENREF_44)^(e)^ | Canada | CCR reg. | 1992-2006 | 2,631 | CNS |  | -0.4% [-1.3;0.5] 10-14 y.o.: −2.3% [−4.4;−0.2] “due to a decrease for astrocytomas” |
| [McKean-Cowdin, et al. 2013](#_ENREF_37) ^(e)^ | USA | SEER, 9 reg. Los Angeles reg. | 1973-2009 | 5,781 | Malign. CNS |  | (SEER classification: pilocytic astrocytomas included)  1.1% [-1.3;3.6] 1973-1982; 14.1% [4.1;25.0] 1983-1986; 0.1% [-0.4;0.6] 1987-2009 |
| [Desandes, et al. 2014](#_ENREF_15) | France | RNTSE reg. | 2000-2008 | 3,886 | Brain |  | -0.2% [-2.9;2.6] p=0.9 |
| [Hung, et al. 2014a](#_ENREF_20) ^(e)^ | Taiwan | TCR reg. | 1995-2009 | 1,449 | CNS |  | 2.1% [0.6; 3.7] |
| [Siegel, et al. 2014](#_ENREF_61) | USA | NPCR(CDC), SEER  (47 reg.) | 2001-2009 | 21,135 1,800 10,533 4,364 3,653 | CNS IIIa IIIb IIIc IIId | 0-19 y.o. | -0.1% [-1.0;0.8]; Boys -0.3% [-1.4;0.9]; Girls 0.1% [-1.1;1.4] 3a. -1.5% [-4.5;1.7]; Boys 0.0% [-3.4;3.5]; Girls -3.2% [-7.1;0.8] 3b. -0.1% [-1.2;0.9]; Boys -0.4% [-1.6;0.9]; Girls 0.1% [-1.6;1.8] 3c. -0.6% [-1.9;0.8]; Boys -1.1% [-2.0;-0.1]; Girls -0.2% [-2.1;2.5] 3d. 0.9% [-0.6;2.4]; Boys 0.3% [-1.4;1.9]; Girls 1.6% [-0.6;3.8] |
| [Ward, et al. 2014](#_ENREF_69) ^(e)^ | USA | SEER, 9 reg. | 1975-2010 | n.a. | Brain  Malign. | 0-19 y.o. | NS (sharp rise in the 1980s on figure 2) |
| [Gittleman, et al. 2015](#_ENREF_17)^(e)^ | USA | CBTRUS  US Cancer Statistics | 2000-2010 | n.a. | CNS |  | 0.6% [0.0;1.1] malignant (2000-2010)  1.6% [-0.3;3.6] non-malignant (2004-2010) |
| [Papathoma, et al. 2015](#_ENREF_47)^(e)^ | Europe SE | 13 reg. Belarus  Serbia  Croatia  Cyprus  Bulgaria  Ukraine  +Portugal, Greece, Romania, Slovenia | 1990-2012  2000-2011  2001-2011  1998-2011  1990-2012  2000-2012 | 1,322  334  250  50  484  2,125 | Malign. CNS |  | -1.4% [-2.4;-0.4] p=0.005 (2003-2012: 2.8% p=0.08) -4.5% [-7.4;-1.4] p=0.0004  -5.5% [-9.2;-1.7] p=0.005 -9.8% [-16.1;-3.0] p=0.01  2.7% [1.4;4.1] p=0.0001 0.1% [-1.0;1.3] p=0.83 (2.1% p=0.005 2000-2008; -5.6% p=0.008 2009-2012)  NS |
| [Isaevska, et al. 2017](#_ENREF_22) ^(e)^ | Italy (Piedmont) | CCRP reg. | 1982-2011  2000-2011 | 728  323 | CNS |  | 1.8% [0.9;2.7]  -0.3% [-4.5;4.2] |
| Other studies^(a)^ | | | | | | | |
| [McNeil, et al. 2002](#_ENREF_43) | USA | SEER, 9 reg. | 1973-1998 | 768 | IIIa. |  | RR=23% between 1973-1977 and 1993-1998 Increase in the proportion of PNET |
| [Dreifaldt, et al. 2004](#_ENREF_16) | Sweden | SCR^1^ reg. | 1960-1998 | 2,569  2,090  1,220 | CNS  Malign.  Gliomas |  | 1.5% [1.0;1.6]  1.0% [0.5;1.6] (3.8% p<0.001 for benign)  1.4% [0.9;2.0] (2.1% p<0.001 grade 1-2;-0.2% p=0.68 grade 3-4) |
| [Johannesen, et al. 2004](#_ENREF_25) | Norway | NCR reg. | 1970-1999 | 1,042 | Brain |  | 2.0% [1.2;2.8%] |
| [Steliarova-Foucher, et al. 2004](#_ENREF_64) | Europe | ACCIS, 63 reg. | 1970-1999 | n.a. | CNS |  | 2.5% p<0.0001 East 0.8% p<0.0001 West |
| [Dalmasso, et al. 2005](#_ENREF_12) | Italy, Piedmont | CCRP reg. | 1967-2001 | 753  276 | CNS  IIIb. |  | 2.3% [1.6;3.1]  3.8% [2.5;5.0] |
| [Kaatsch, et al. 2006](#_ENREF_28) | Europe | ACCIS, 33 reg. | 1978-1997 | 17,057 | CNS |  | 1.7% p<0.0001 |
| [Spix, et al. 2008](#_ENREF_62) | Germany | GCCR reg. West East | 1987-2004  1991-2004 | 5,155 775 | CNS  CNS | 0-19 y.o. | 1.1% [0.6;1.6] West  5.5% [4.6;7.4] East |
| [Bao, et al. 2010](#_ENREF_5) | Shanghai | SCR2 reg. | 1973-2005 | 585 | CNS | (ICD-9) | -0.5% [-2.2;1.3] boys; -0.3% [-2.0;1.5] girls |
| [Rosychuk, et al. 2010](#_ENREF_54) | Canada | ACR | 1982-2004 | 568 | CNS | 0-19 y.o. | incidence rate=2.1 per 100,000 in 1983/1984; 4.2 per 100,000 in 2003/2004. "Our data suggests an emerging trend with the latter few years having a seemingly higher standardized incidence rate than earlier years. " |

^(a)^ The first part of the table includes the results from 1) studies based on a recent time period (starting in 1990 or after), and 2) studies based on a time period starting before 1990, with consideration of temporal variations in the most recent years (mainly with nonlinear modeling). The second part of the table includes the studies that considered a log-linear temporal trend on a long time period starting before 1990. Improvements in diagnostic methods and cancer registration have certainly played a role in the increases reported in the latter studies (cf discussion section in the manuscript).

^(b)^ Reg.: registry; ACCIS: Automated Cancer Information System; ACR: Alberta Cancer Registry; APCR: Australian Paediatric Cancer Registry; CBTRUS: Central Brain Tumor Registry of US; CCR: Canadian Cancer Registry; CCRP: Childhood Cancer Registry of Piedmont; CDC: Centers for Disease Control and Prevention; GCCR: German Childhood Cancer Registry; NCR: Norwegian Cancer Registry; NPCR: National Program of Cancer Registries; NRCT: National Registry of Childhood Tumors; NSPHO: Nordic Society of Paediatric Haematology and Oncology; POGO: Pediatric Oncology Group of Ontario; RNTSE: French National Registry of Childhood Solid Tumors; SCR^1^: Swedish Cancer Registry; SCR^2^: Shanghai Cancer Registry; SEER: Surveillance, Epidemiology, and Results Program; TCR: Taiwan Cancer Registry period considered for time-trend; ^(c)^ Number of cases (N) and diagnostic group (0-14 y.o. if not otherwise specified); (d) CNS: central nervous system tumor; Malign: malignant CNS tumor; IIIa. Ependymomas and choroid plexus tumors; IIIb: astrocytomas; IIIc. Intracranial and intraspinal embryonal tumors; IIId. Other gliomas; IIIe. Other specified intracranial and intraspinal neoplasms; IIIf. Unspecified intracranial and intraspinal neoplasms; n.a.: not available; ^(e)^ nonlinear variation was considered

AAPC: Average Annual Percent Change; NS: Not Significant according to the authors.

**Supplementary table 4** Time variation in the incidence of childhood peripheral nervous cell (PNC) tumors - main results of the papers published since 2000^(a)^

| **Reference** | **Country** | **Source of data**^(b)^ | **Period**^(c)^ |  | **N**^(d)(e)^ |  | **Main results (AAPC if not otherwise specified)** ^(e)^ |
| --- | --- | --- | --- | --- | --- | --- | --- |
| Studies with results on recent temporal trend^(a)^ | | | | | | | |
| [Maule, et al. 2006](#_ENREF_36) ^(f)^ | Italy, Piedmont | CCRP reg. | 1967-2001 | 254 | NB |  | Increase (on figure 2) |
| [Linabery and Ross 2008](#_ENREF_34) ^(f)^ | USA | SEER, 13 reg. | 1992-2004 | 1,107 | NB | 0-19 y.o. | -0.6% [-2.9;1.7] |
| [Spix, et al. 2008](#_ENREF_62) | Germany | GCCR reg.  West  East | 1987-2004  1991-2004 | 2,106 228 | PNC  PNC |  | 0.1% [-1.5;1.8]  2.3% [-0.8;5.5] |
| [Baade, et al. 2010](#_ENREF_4) ^(f)^ | Australia | APCR reg. | 1983-2006 | 869 | PNC |  | 0.2% [-1.1;1.4] |
| [Kroll, et al. 2012](#_ENREF_31) ^(f)^ | Great Britain | NRCT reg. | 1966-2005 | 3,552 | PNC |  | 0.6% [0.4;0.9] stable since 1987 |
| [Mitra, et al. 2012](#_ENREF_44) ^(f)^ | Canada | CCR reg. | 1992-2006 | 960 | NB |  | −0.2% [−1.8;1.5] |
| [Hung, et al. 2014a](#_ENREF_20) ^(f)^ | Taiwan | TCR reg. | 1995-2009 | 587 | PNC | 0-19 y.o. | 1.7% [-0.5, 4.1];  Boys: 3.0% [-0.1, 6.2]; Girls: 0.1% [-3.5, 3.8] |
| [Siegel, et al. 2014](#_ENREF_61) | USA | NPCR(CDC), SEER (47 reg.) | 2001-2009 | 5,870 | PNC | 0-19 y.o. | -1.2% [-3.0;0.8] Boys: -0.5% [-2.9;2.0]; Girls: -2.0% [-4.0;0.1] |
| [Ward, et al. 2014](#_ENREF_69) ^(f)^ | USA | SEER, 9 reg. | 1975-2010 | n.a. | NB | 0-19 y.o. | NS |
| [Tulla, et al. 2015](#_ENREF_67) | Germany | GCCR reg. | 1991-2012 | 2,989 | NB |  | 0.2% [0.4;-0.8] |
| [Isaevska, et al. 2017](#_ENREF_22) ^(f)^ | Italy (Piedmont) | CCRP reg. | 1976-2011  2000-2011 | 286  114 | NB |  | 1.2% [0.2;2.1] (2.5% [0.7;4.2] for < 1 y.o.)  -2.4% [-7.0;2.5]  *1990-2011: 1.2% [0.2;2.1] (2.5% [0.7;4.2] for < 1 y.o.)* |
| Other studies^(a)^ | | | | | | | |
| [Dreifaldt, et al. 2004](#_ENREF_16) | Sweden | SCR reg. | 1960-1998 | 459 | PNC |  | 1.6% [0.8;2.4] |
| [Steliarova-Foucher, et al. 2004](#_ENREF_64) | Europe | ACCIS, 63 reg. | 1970-1999 | n.a. | NB |  | 2.0% p<0.0001 |
| [Kaatsch, et al. 2006](#_ENREF_28) | Europe | ACCIS, 33 reg. | 1978-1997 | 5,580 | PNC |  | 1.7% p<0.0001 |
| [Dalmasso, et al. 2005](#_ENREF_12) | Italy (NW,  Piedmont) | CCRP reg. | 1967-2001 | 254 | PNC |  | 2.3% [1.0;3.5] |

^(a)^ The first part of the table includes the results from 1) studies based on a recent time period (starting in 1990 or after), and 2) studies based on a time period starting before 1990, with consideration of temporal variations in the most recent years (mainly with nonlinear modeling). The second part of the table includes the studies that considered a log-linear temporal trend on a long time period starting before 1990. Improvements in diagnostic methods and cancer registration have certainly played a role in the increases reported in the latter studies (cf discussion section in the manuscript). ^(b)^ Reg.: registry; ACCIS: Automated Cancer Information System; APCR: Australian Paediatric Cancer Registry; CCR: Canadian Cancer Registry; CCRP: Childhood Cancer Registry of Piedmont; CDC: Centers for Disease Control and Prevention; GCCR: German Childhood Cancer Registry; NPCR: National Program of Cancer Registries; NRCT: National Registry of Childhood Tumors; SCR: Swedish Cancer Registry; SEER: Surveillance, Epidemiology, and Results Program; TCR: Taiwan Cancer Registry. ^(c)^ period considered for time-trend; ^(d)^ Number of cases (N) and diagnostic group (0-14 y.o. if not otherwise specified); n.a.: not available; ^(e)^ NB: neuroblastomas; PNC tum.: peripheral nervous cell tumors; ^(f)^ nonlinear variation was considered. AAPC: Average Annual Percent Change; NS: Not Significant according to the authors.

**Supplementary table 5** Time variation in the incidence of childhood retinoblastomas - main results of the papers published since 2000^(a)^

| **Reference** | **Country** | **Source of data**^(b)^ | **Period**^(c)^ | **N**^(d)^ | | **Main results (AAPC if not otherwise specified)** |
| --- | --- | --- | --- | --- | --- | --- |
| Studies with results on recent temporal trend^(a)^ | | | | | | |
| [Linabery and Ross 2008](#_ENREF_34)^(e)^ | USA | SEER, 13 reg. | 1992-2004 | 514 | 0-19 y.o. | 0.3% [-1.5;2.1] |
| [Spix, et al. 2008](#_ENREF_62) | Germany | GCCR reg. West  East | 1987-2004  1991-2004 | 604 41 |  | -0.8% [-2.5;1.0] insufficient data |
| [Baade, et al. 2010](#_ENREF_4) ^(e)^ | Australia | APCR reg. | 1983-2006 | 357 |  | 0.1% [-1.1;1.4] |
| [Kroll, et al. 2012](#_ENREF_31) ^(e)^ | Great Britain | NRCT reg. | 1966-2005 | 1,598 |  | 0.6% [0.2;1.1] step in 1993 |
| [Mitra, et al. 2012](#_ENREF_44) ^(e)^ | Canada | CCR reg. | 1992-2006 | 322 |  | −2.6% [−4.7;−0.4] |
| [Hung, et al. 2014a](#_ENREF_20) ^(e)^ | Taiwan | TCR reg. | 1995-2009 | 228 | 0-19 y.o. | -0.1% [-2.8, 2.8] |
| [Siegel, et al. 2014](#_ENREF_61) | USA | NPCR (CDC, SEER 47 reg.) | 2001-2009 | 2,169 | 0-19 y.o. | -0.2% [-1.8;1.5];  Boys: -1.6% [-3.6;0.3]; Girls: 1.4% [-1.1;3.9] |
| [Ward, et al. 2014](#_ENREF_69) ^(e)^ | USA | SEER, 9 reg. | 1975-2010 | n.a. | 0-19 y.o. | NS |
| [Tulla, et al. 2015](#_ENREF_67) | Germany | GCCR reg. | 1991-2012 | 879 |  | 0.5% [-0.5;1.6] |
| [Isaevska, et al. 2017](#_ENREF_22) ^(e)^ | Italy (Piedmont) | CCRP reg. | 1976-2011 | 81 |  | less than 5 cases/year on average |
| Other studies^(a)^ | | | | | | |
| [Dreifaldt, et al. 2004](#_ENREF_16) | Sweden | SCR reg. | 1960-1998 | 254 |  | 0.3% [-1.2;1.7] |
| [Seregard, et al. 2004](#_ENREF_59) | Sweden  Finland | SCR reg.  FCR reg. | 1958-1998 | 465 | 0-5 y.o. | Linear slope in incidence rate (/100000)  Diag: 0.02 [-0.61;0.95] p=0.67 (increase suggested after 1990)  Birth cohort: 0.03 [-0.00;0.08] p=0.23 (increase suggested after 1988) |
| [Steliarova-Foucher, et al. 2004](#_ENREF_64) | Europe | ACCIS, 63 reg. | 1970-1999 | n.a. |  | 1.1% p=0.018 |
| [Dalmasso, et al. 2005](#_ENREF_12) | Italy  (NW, Piedmont) | CCRP reg. | 1967-2001 | 85 |  | 1.1% [-0.9;3.3] |
| [Kaatsch, et al. 2006](#_ENREF_28) | Europe | ACCIS, 33 reg. | 1978-1997 | 1,995 |  | 0.5% NS |
| [Broaddus, et al. 2009](#_ENREF_8) | USA | SEER, 9 reg. | 1975-2004 | 658 | 0-4 y.o. | NS |

^(a)^ The first part of the table includes the results from 1) studies based on a recent time period (starting in 1990 or after), and 2) studies based on a time period starting before 1990, with consideration of temporal variations in the most recent years (mainly with nonlinear modeling). The second part of the table includes the studies that considered a log-linear temporal trend on a long time period starting before 1990. Improvements in diagnostic methods and cancer registration have certainly played a role in the increases reported in the latter studies (cf discussion section in the manuscript). ^(b)^ Reg.: registry; ACCIS: Automated Cancer Information System; APCR: Australian Paediatric Cancer Registry; CCR: Canadian Cancer Registry; CCRP: Childhood Cancer Registry of Piedmont; CDC: Centers for Disease Control and Prevention; FCR: Finnish Cancer registry; GCCR: German Childhood Cancer Registry; NPCR: National Program of Cancer Registries; NRCT: National Registry of Childhood Tumors; SCR: Swedish Cancer Registry; SEER: Surveillance, Epidemiology, and Results Program; TCR: Taiwan Cancer Registry. ^(c)^ Period considered for time-trend. (d) Number of cases (0-14 y.o. if not otherwise specified); n.a.: not available; (e) nonlinear variation was considered. AAPC: Average Annual Percent Change; NS: Not Significant according to the authors.

**Supplementary table 6** Time variation in the incidence of childhood renal tumors - main results of the papers published since 2000^(a)^

| **Reference** | **Country** | **Source of data**^(b)^ | **Period**^(c)^ | **N**^(d)(e)^ | | | **Main results (AAPC if not otherwise specified)** |
| --- | --- | --- | --- | --- | --- | --- | --- |
| Studies with results on recent temporal trend^(a)^ | | | | | | | |
| [Linabery and Ross 2008](#_ENREF_34) ^(f)^ | USA | SEER, 13 reg. | 1992-2004 | 804 | Wilm’s tumor | 0-19 y.o. | -2.1% [-4.6;0.4] |
| [Spix, et al. 2008](#_ENREF_62) | Germany | GCCR reg. West  East | 1987-2004  1991-2004 | 1546 201 |  |  | 0.6% [-0.4;1.6]  1.8% [-1.5;5.2] |
| [Baade, et al. 2010](#_ENREF_4) ^(f)^ | Australia | APCR reg. | 1983-2006 | 735 |  |  | 0.4% [0.7,1.6] |
| [Kroll, et al. 2012](#_ENREF_31) ^(f)^ | Great Britain | NRCT reg. | 1966-2005 | 3,214 |  |  | 0.7% [0.4;1.0] step in 2000 |
| [Mitra, et al. 2012](#_ENREF_44) ^(f)^ | Canada | CCR reg. | 1992-2006 | 757 |  |  | -1.3% [-3.2;0.7] |
| [Hung, et al. 2014a](#_ENREF_20) ^(f)^ | Taiwan | TCR reg. | 1995-2009 | 243 |  | 0-19 y.o. | -1.1% [-4.0;1.9] |
| [Ward, et al. 2014](#_ENREF_69) ^(f)^ | USA | SEER, 9 reg. | 1975-2010 | n.a. | Wilm's tumor | 0-19 y.o. | NS |
| [Tulla, et al. 2015](#_ENREF_67) | Germany | GCCR reg. | 1991-2012 | 2,160 | Nephrob. |  | 0.5% [-0.1;1.2] |
| [Isaevska, et al. 2017](#_ENREF_22) ^(e)^ | Italy (Piedmont) | CCRP reg. | 1976-2011 | 167 |  |  | less than 5 cases/year on average |
| Other studies^(a)^ | | | | | | | |
| [Dreifaldt, et al. 2004](#_ENREF_16) | Sweden | SCR reg. | 1960-1998 | 547 |  |  | 0.3% [-0.5;1.1] |
| [Steliarova-Foucher, et al. 2004](#_ENREF_64) | Europe | ACCIS, 63 reg. | 1970-1999 | n.a. |  |  | 1.1% p=0.017 |
| [Dalmasso, et al. 2005](#_ENREF_12) | Italy (NW, Piedmont) | CCRP reg. | 1967-2001 | 161 |  |  | 1.1% [-0.4;2.7] |
| [Kaatsch, et al. 2006](#_ENREF_28) | Europe | ACCIS, 33 reg. | 1978-1997 | 4549 |  |  | 0.8% p<0.01 |
| [Siegel, et al. 2014](#_ENREF_61) | USA | NPCR (CDC), SEER, 47 reg. | 2001-2009 | 4,697 4,248 426 | Renal tumor Nephrob. Carcinoma | 0-19 y.o. | 0.5% [-0.3;1.3]; Boys 0.0% [-1.5;1.5]; Girls 1.1% [-1.3;3.2] 0.0% [-0.7;0.8]; Boys -0.4% [-1.9;1.1]; Girls 0.4% [-1.7;2.5] -5.4% [2.8;8.1]; Boys 4.2% [1.4;7.0]; Girls n.a. |

^(a)^ The first part of the table includes the results from 1) studies based on a recent time period (starting in 1990 or after), and 2) studies based on a time period starting before 1990, with consideration of temporal variations in the most recent years (mainly with nonlinear modeling). The second part of the table includes the studies that considered a log-linear temporal trend on a long time period starting before 1990. Improvements in diagnostic methods and cancer registration have certainly played a role in the increases reported in the latter studies (cf discussion section in the manuscript). ^(b)^ Reg.: registry; ACCIS: Automated Cancer Information System; APCR: Australian Paediatric Cancer Registry; CCR: Canadian Cancer Registry; CCRP: Childhood Cancer Registry of Piedmont; CDC: Centers for Disease Control and Prevention; GCCR: German Childhood Cancer Registry; NPCR: National Program of Cancer Registries; NRCT: national Registry of Childhood Tumors; SCR: Swedish Cancer Registry; SEER: Surveillance, Epidemiology, and Results Program; TCR: Taiwan Cancer Registry. ^(c)^ period considered for time-trend; ^(d)^ Number of cases (N) and diagnostic group (0-14 y.o. if not otherwise specified); n.a.: not available; ^(e)^ Nephrob.: nephroblastoma; ^(f)^ nonlinear variation was considered.

AAPC: Average Annual Percent Change; NS: Not Significant according to the authors

**Supplementary table 7** Time variation in the incidence of childhood hepatic tumors - main results of the papers published since 2000^(a)^

| **Reference** | **Country** | **Source of data**^(b)^ | **Period**^(c)^ | **N**^(d)(e)^ | | | **Main results (AAPC if not otherwise specified)** |
| --- | --- | --- | --- | --- | --- | --- | --- |
| Studies with results on recent temporal trend^(a)^ | | | | | | | |
| [Linabery and Ross 2008](#_ENREF_34) ^(e)^ | USA | SEER, 13 reg. | 1992-2004 | 216 |  | 0-19 y.o. | 4.3% [0.2;8.7]; 10.4% [4.9;16.3] < 1 y.o.; 4.8% [0.9;8.8] 1-4 y.o. |
| [Spix, et al. 2008](#_ENREF_62) | Germany | GCCR reg. West  East | 1987-2004  1991-2004 | 270 22 |  |  | 1.5% [-0.9;3.9]  insufficient data |
| [Baade, et al. 2010](#_ENREF_4) ^(e)^ | Australia | APCR reg. | 1983-2006 | 174 |  |  | 3.3% [0.8;5.9] |
| [Kroll, et al. 2012](#_ENREF_31) ^(e)^ | Great Britain | NRCT reg. | 1966-2005 | 483 |  |  | 2.5% [1.7;3.3] steps in 1984 and 2001 |
| [Mitra, et al. 2012](#_ENREF_44) ^(e)^ | Canada | CCR reg. | 1992-2006 | 200 |  |  | 1.6% [−0.8;4.0] |
| [Hung, et al. 2014a](#_ENREF_20) ^(e)^ | Taiwan | TCR reg. | 1995-2009 | 424 |  | 0-19 y.o. | -2.2% [-4.6;0.3]; Boys: -3.2% [-5.7;-0.6] |
| [Siegel, et al. 2014](#_ENREF_61) | USA | NPCR (CDC), SEER, 47 reg. | 2001-2009 | 1,477 1,050 411 | Hepatob.  Carcin. | 0-19 y.o. | 1.7% [-1.7;5.3]; Boys 2.8% [-1.4;7.2]; Girls 0.1% [-4.1;4.4] 1.6% [-2.2;5.6]; Boys 2.4% [-1.9;6.8]; Girls 0.3% [-4.8;5.7] 2.1% [-0.7;4.9]; Boys 3.8% [-0.7;8.5]; Girls 0.2% [-3.8;4.4] |
| [Tulla, et al. 2015](#_ENREF_67) | Germany | GCCR reg. | 1991-2012 | 351 | Hepatob. |  | 4.6% [2.9;6.4] |
| [Isaevska, et al. 2017](#_ENREF_22) ^(e)^ | Italy (Piedmont) | CCRP reg. | 1976-2011 | 42 |  |  | less than 5 cases/year on average |
| Other studies^(a)^ | | | | | | | |
| [Dreifaldt, et al. 2004](#_ENREF_16) | Sweden | SCR1 reg. | 1960-1998 | 100 |  |  | 2.6% [2.0;3.2] |
| [Steliarova-Foucher, et al. 2004](#_ENREF_64) | Europe | ACCIS, 63 reg. | 1970-1999 | n.a. |  |  | 1.0% p=0.027 |
| [Dalmasso, et al. 2005](#_ENREF_12) | Italy (NW, Piedmont) | CCRP reg. | 1967-2001 | 33 |  |  | 0.0% [-3.3;3.5] |
| [Kaatsch, et al. 2006](#_ENREF_28) | Europe | ACCIS, 33 reg. | 1978-1997 | 749 |  |  | 0.8% p>0.05 |

^(a)^ The first part of the table includes the results from 1) studies based on a recent time period (starting in 1990 or after), and 2) studies based on a time period starting before 1990, with consideration of temporal variations in the most recent years (mainly with nonlinear modeling). The second part of the table includes the studies that considered a log-linear temporal trend on a long time period starting before 1990. Improvements in diagnostic methods and cancer registration have certainly played a role in the increases reported in the latter studies (cf discussion section in the manuscript). ^(b)^ Reg.: registry; ACCIS: Automated Cancer Information System; APCR: Australian Paediatric Cancer Registry; CCR: Canadian Cancer Registry; CCRP: Childhood Cancer Registry of Piedmont; CDC: Centers for Disease Control and Prevention; GCCR: German Childhood Cancer Registry; NPCR: National Program of Cancer Registries; NRCT: National Registry of Childhood Tumors; SCR: Swedish Cancer Registry; SEER: Surveillance, Epidemiology, and Results Program; TCR: Taiwan Cancer Registry. ^(c)^ period considered for time-trend; ^(d)^ Number of cases (N) and diagnostic group (0-14 y.o. if not otherwise specified); n.a.: not available; Hepatob.: hepatoblastoma; Carcin.: carcinoma; ^(e)^ nonlinear variation was considered

AAPC: Average Annual Percent Change.

**Supplementary table 8** Time variation in the incidence of childhood bone tumors - main results of the papers published since 2000^(a)^

| **Reference** | **Country** | **Source of data**^(b)^ | **Period**^(c)^ | **N**^(d)^ | | | **Main results (AAPC if not otherwise specified)** |
| --- | --- | --- | --- | --- | --- | --- | --- |
| Studies with results on recent temporal trend^(a)^ | | | | | | | |
| [Linabery and Ross 2008](#_ENREF_34)^(e)^ | USA | SEER, 13 reg. | 1992-2004 | 660  327 | Osteosarc  Ewing’s tum. | 0-19 y.o. | 0.2% [-1.4;1.8]; 0.2% [-2.5;3.0] 10-14 y.o. (267 cases)  -3.4% [-7.0;0.3]; -4.3% [-7.7;-0.7] 10-14 y.o. (109 cases) |
| [Spix, et al. 2008](#_ENREF_62) | Germany | GCCR reg.  West  East | 1987-2004  1991-2004 | 1,104 197 |  |  | -0.1% [-1.3;1.0]  0.6% [-2.9;4.3] |
| [Baade, et al. 2010](#_ENREF_4) ^(e)^ | Australia | APCR reg. | 1983-2006 | 602 |  |  | 0.3% [-0.8;1.3] |
| [Kroll, et al. 2012](#_ENREF_31) ^(e)^ | Great Britain | NRCT reg. | 1966-2005 | 2,427 |  |  | 0.5% [0.2;0.9] step in 1972  (stability until 2001, heterogeneous over 2002-2005) |
| [Mitra, et al. 2012](#_ENREF_44) ^(e)^ | Canada | CCR reg. | 1992-2006 | 598 |  |  | −1.2% [−2.8;0.5] |
| [Hung, et al. 2014a](#_ENREF_20) ^(e)^ | Taiwan | TCR reg. | 1995-2009 | 705 |  | 0-19 y.o. | 0.1% [-1.8;2.0] |
| [Hung, et al. 2014b](#_ENREF_21)^(e)^ | Taiwan | TCR reg. | 2003-2010 | 449 300 54 | Osteosarc  Ewing’s tum. | 0-24 y.o. | 0.6% [-3.5;4.8] 3.3% [-0.5;7.2] 4.5% [-12.8;25.2] |
| [Siegel, et al. 2014](#_ENREF_61) | USA | NPCR (CDC),  SEER (47 reg.) | 2001-2009 | 6,285 3,546 2,054 | Osteosarc. Ewing's tum. | 0-19 y.o. | -0.6% [-1.3;0.2]; Boys -0.5% [-2.1;1.1]; Girls -0.7% [-1.8;0.5] -0.4% [-1.7;1.0]; Boys -0.9% [-2.7;0.9]; Girls 0.3% [-2.3;3.1] -1.0% [-3.2;1.4]; Boys -0.3% [-4.0;3.7]; Girls -1.9% [-3.8;0.0] |
| [Ward, et al. 2014](#_ENREF_69) ^(e)^ | USA | SEER, 9 reg. | 1975-2010 | n.a. |  | 0-19 y.o. | NS |
| [Isaevska, et al. 2017](#_ENREF_22) ^(e)^ | Italy (Piedmont) | CCRP reg. | 1976-2011  2000-2011 | 190  51 |  |  | 0.0% [-4.1;4.4]  less than 5 cases/year on average |
| Other studies^(a)^ | | | | | | | |
| [Dreifaldt, et al. 2004](#_ENREF_16) | Sweden | SCR reg. | 1960-1998 | 360 |  |  | 0.2% [-0.8;1.3] |
| [Steliarova-Foucher, et al. 2004](#_ENREF_64) | Europe | ACCIS, 63 reg. | 1970-1999 | n.a. |  |  | 0.4% p=0.023 |
| [Dalmasso, et al. 2005](#_ENREF_12) | Italy (NW, Piedmont) | CCRP reg. | 1967-2001 | 179 |  |  | -0.7% [-2.3;0.9] |
| [Kaatsch, et al. 2006](#_ENREF_28) | Europe | ACCIS, 33 reg. | 1978-1997 | 3,692 |  |  | -0.3% p>0.05 |
| [Stiller, et al. 2006](#_ENREF_65) | Europe | ACCIS, 33 reg. | 1978-1997 | 3,429 1,766 1,430 | Osteosarc. Ewing's tum. |  | -0.1% [-0.8;0.5] 0.4% [-0.5;1.4] -1.0% [-2.0;0.0] |

^(a)^ The first part of the table includes the results from 1) studies based on a recent time period (starting in 1990 or after), and 2) studies based on a time period starting before 1990, with consideration of temporal variations in the most recent years (mainly with nonlinear modeling). The second part of the table includes the studies that considered a log-linear temporal trend on a long time period starting before 1990. Improvements in diagnostic methods and cancer registration have certainly played a role in the increases reported in the latter studies (cf discussion section in the manuscript). ^(b)^ Reg.: registry; ACCIS: Automated Cancer Information System; APCR: Australian Paediatric Cancer Registry; CCR: Canadian Cancer Registry; CCRP: Childhood Cancer Registry of Piedmont; CDC: Centers for Disease Control and Prevention; GCCR: German Childhood Cancer Registry; NPCR: National Program of Cancer Registries; NRCT: National Registry of Childhood Tumors; SCR: Swedish Cancer Registry; SEER: Surveillance, Epidemiology, and Results Program; TCR: Taiwan Cancer Registry. ^(c)^ period considered for time-trend; ^(d)^ Number of cases (N) and diagnostic group (0-14 y.o. if not otherwise specified); n.a.: not available; Ewing’s tum.: Ewing’s tumor; Osteosarc.: osteosarcoma; ^(e)^ nonlinear variation was considered

AAPC: Average Annual Percent Change; NS: Not Significant according to the authors

**Supplementary table** **9** Time variation in the incidence of childhood soft tissue and other extraosseous sarcomas - main results of the papers published since 2000^(a)^

| **Reference** | **Country** | **Source of data**^(b)^ | **Period**^(c)^ | **N**^(d)(e)^ | | | **Main results (AAPC if not otherwise specified)** ^(e)^ |
| --- | --- | --- | --- | --- | --- | --- | --- |
| Studies with results on recent temporal trend^(a)^ | | | | | | | |
| [Linabery and Ross 2008](#_ENREF_34) ^(f)^ | USA | SEER, 13 reg. | 1992-2004 | 643  925 | RMS  Other STS | 0-19 y.o. | 0.8% [-1.8;3.4]  0.8% [-1.3;2.9] |
| [Spix, et al. 2008](#_ENREF_62) | Germany | GCCR reg.  West  East | 1987-2004  1991-2004 | 1,647 244 | STS  STS |  | 1.3% [0.3;2.3]  -0.6% [-3.7;2.6] |
| [Baade, et al. 2010](#_ENREF_4) ^(f)^ | Australia | APCR reg. | 1983-2006 | 820 | STS |  | -0.2% [-1.4;1.1] |
| [Kroll, et al. 2012](#_ENREF_31) ^(f)^ | Great Britain | NRCT reg. | 1966-2005 | 3,549 | STS |  | 1.6% [1.3;2.3] last step in 1984 |
| [Mitra, et al. 2012](#_ENREF_44) ^(f)^ | Canada | CCR reg. | 1992-2006 | 816 | STS |  | −1.4% [−3.6;0.8] |
| [Hung, et al. 2014a](#_ENREF_20) ^(f)^ | Taiwan | TCR reg. | 1995-2009 | 933 | STS | 0-19 y.o. | 2.5% [1.1, 3.9] |
| [Siegel, et al. 2014](#_ENREF_61) | USA | NPCR (CDC), SEER 47 reg. | 2001-2009 | 8,419 3,299 | STS RMS | 0-19 y.o. | 0.3% [-0.4;1.0]; Boys 0.0% [-1.0;1.1]; Girls 0.6% [-1.1;2.4] -0.7% [-3.0;1.7]; Boys -1.1% [-3.0;1.0]; Girls -0.1% [-4.2;4.2] |
| [Ward, et al. 2014](#_ENREF_69) ^(f)^ | USA | SEER, 9 reg. | 1975-2010 | n.a. | RMS | 0-19 y.o. | NS |
| [Isaevska, et al. 2017](#_ENREF_22) ^(e)^ | Italy (Piedmont) | CCRP reg. | 1976-2011  2000-2011 | 206  64 |  |  | 0.5% [-0.7;1.7]  less than 5 cases/year on average |
| Other studies^(a)^ | | | | | | | |
| [Dreifaldt, et al. 2004](#_ENREF_16) | Sweden | SCR reg. | 1960-1998 | 510 | STS |  | 0.1% [-0.8;1.0] |
| [Steliarova-Foucher, et al. 2004](#_ENREF_64) | Europe | ACCIS, 63 reg. | 1970-1999 | n.a. | STS |  | 1.8% p<0.0001 |
| [Dalmasso, et al. 2005](#_ENREF_12) | Italy (NW, Piedmont) | CCRP reg. | 1967-2001 | 195 | STS |  | 1.4% [0.0;2.9] |
| [Kaatsch, et al. 2006](#_ENREF_28) | Europe | ACCIS, 32 reg. | 1978-1997 | 5,111 | STS |  | 1.8% p<0.0001 |
| [Pastore, et al. 2006](#_ENREF_48) | Europe | ACCIS, 32 reg. | 1978-1997 | 5,111  2,940  721  10  1,084 | STS  RMS  Fibrosarc.  Kaposi Sarc.  Other spec. |  | 1.8% p<0.0001 RMS: 1.3% p<0.0001 (stability over 1988-1997 suggested by table 7) 0.01% p=0.99  Insufficient data  2.8% p<0.0001 |
| [Weihkopf, et al. 2008](#_ENREF_70) | Germany | GCCR reg. | 1985-2004 | 2,061  1,202 | STS  RMS |  | 0.4% [-0.4;1.2]  0.1% [-0.9;1.1] |
| [Ognjanovic, et al. 2009](#_ENREF_46) | USA | SEER, 9 reg. | 1975-2005 | 987 | RMS | 0-19 y.o. | 0.5% [-0.3;1.3] Embryonal RMS -0.4% [-1.5;0.7]  Alveolar RMS 4.2% [2.6;5.8] |

^(a)^ The first part of the table includes the results from 1) studies based on a recent time period (starting in 1990 or after), and 2) studies based on a time period starting before 1990, with consideration of temporal variations in the most recent years (mainly with nonlinear modeling). The second part of the table includes the studies that considered a log-linear temporal trend on a long time period starting before 1990. Improvements in diagnostic methods and cancer registration have certainly played a role in the increases reported in the latter studies (cf discussion section in the manuscript). ^(b)^ Reg.: registry; ACCIS: Automated Cancer Information System; APCR: Australian Paediatric Cancer Registry; CCR: Canadian Cancer Registry; CCRP: Childhood Cancer Registry of Piedmont; CDC: Centers for Disease Control and Prevention; GCCR: German Childhood Cancer Registry; NPCR: National Program of Cancer Registries; NRCT: National Registry of Childhood Tumors; SCR: Swedish Cancer Registry; SEER: Surveillance, Epidemiology, and Results Program; TCR: Taiwan Cancer Registry. ^(c)^ period considered for time-trend; ^(d)^ Number of cases (N) and diagnostic group (0-14 y.o. if not otherwise specified); n.a.: not available; ^(e)^ Fibrosarc.: fibrosarcoma; sarc.: sarcoma; Other spec.: other specified; STS: soft tissue sarcoma; RMS: rhabdomyosarcoma; ^(f)^ nonlinear variation was considered;

AAPC: Average Annual Percent Change; NS: Not Significant according to the authors

**Supplementary table 10** Time variation in the incidence of childhood germ cell tumors (GCT) - main results of the papers published since 2000^(a)^

| **Reference** | **Country** | **Source of data**^(b)^ | **Period**^(c)^ | **N**^(d)^ | | | **Main results (AAPC if not otherwise specified)** |
| --- | --- | --- | --- | --- | --- | --- | --- |
| Studies with results on recent temporal trend^(a)^ | | | | | | | |
| [Linabery and Ross 2008](#_ENREF_34) ^(e)^ | USA | SEER, 13 reg. | 1992-2004 | 1,607 | GCT | 0-19 y.o. | 0.8% [-0.7;2.4] |
| [Spix, et al. 2008](#_ENREF_62) | Germany | GCCR reg.  West  East | 1987-2004  1991-2004 | 880  117 | GCT  GCT |  | -0.2% [-1.5;1.1]  -1.1% [-5.6;3.5] |
| [Baade, et al. 2010](#_ENREF_4) ^(e)^ | Australia | APCR reg. | 1983-2006 | 511  253  258 | GCT Boys  Girls |  | 2.3% [0.9;3.7]  2.6% [0.8;4.5]  2.0% [0.2;3.9] |
| [Kroll, et al. 2012](#_ENREF_31) ^(e)^ | Great Britain | NRCT reg. | 1966-2005 | 1,224 | Non CNS |  | 1.1% [0.6;1.6] step in 1981 |
| [Mitra, et al. 2012](#_ENREF_44) ^(e)^ | Canada | CCR reg. | 1992-2006 | 439  201  237 | GCT Boys  Girls |  | −0.4% [−2.2;1.4] −1.1% [−3.8;1.6]  0.0% [−3.6;3.7] |
| [Hung, et al. 2014a](#_ENREF_20) ^(e)^ | Taiwan | TCR reg. | 1995-2009 | 1,267 | GCT | 0-19 y.o. | 2.3% [0.8; 3.9]; Boys: 3.2% [1.2; 5.3]; Girls: 1.2% [-1.3; 3.8] |
| [Siegel, et al. 2014](#_ENREF_61) | USA | NPCR (CDC), SEER, 47 reg. | 2001-2009 | 7,814 1,133 1,046 5,142 | GCT Xa Xb Xc | 0-19 y.o. | 0.7% [-0.5;2.0]; Boys 1.1% [-0.7;2.6]; Girls 0.5% [-1.3;2.2] 0.8% [-1.6;3.2]; Boys -1.7% [-1.3;4.8]; Girls -1.3% [-7.3;5.0] -2.0% [-3.9;-0.2]; Boys 1.7% [-3.8;0.4]; Girls -2.3% [-4.5;-0.1] 1.1% [-0.6;2.9]; Boys 1.0% [-1.1;3.2]; Girls 1.5% [-0.5;3.5] |
| [Ward, et al. 2014](#_ENREF_69) ^(e)^ | USA | SEER, 9 reg. | 1975-2010 | n.a. | GCT | 0-19 y.o. | testicular GCT: 1.2% ovarian GCT: NS |
| [Kaatsch, et al. 2015](#_ENREF_26) ^(e)^ | Germany | GCCR reg. | 1987-2011 | 1,366  380  406  580 | GCT  Xa  Xb  Xc |  | 0.3% [-0.4;1.1]  0.2% [-1.3;1.7]  -0.1% [-1.4;1.3]  0.7% [-0.5;1.9] |
| [Isaevska, et al. 2017](#_ENREF_22) ^(e)^ | Italy (Piedmont) | CCRP reg. | 1976-2011 | 94 |  |  | less than 5 cases/year on average |
| Other studies^(a)^ | | | | | | | |
| [Dreifaldt, et al. 2004](#_ENREF_16) | Sweden | SCR reg. | 1960-1998 | 249 | GCT |  | 1.2% [0.2;2.2] |
| [Steliarova-Foucher, et al. 2004](#_ENREF_64) | Europe | ACCIS, 63 reg. | 1970-1999 | n.a. | GCT |  | 2.3% p<0.0001 |
| [Dalmasso, et al. 2005](#_ENREF_12) | Italy (NW, Piedmont) | CCRP reg. | 1967-2001 | 86 | GCT |  | 3.0% [0.8;5.3] |
| [Kaatsch, et al. 2006](#_ENREF_28) | Europe | ACCIS, 32 reg. | 1978-1997 | 2,555 | GCT |  | 3.0% [0.8;5.3] |
| [Walsh, et al. 2006](#_ENREF_68) | USA | SEER, ~11 reg. | 1973-2000 | 131 | Testicular |  | 1.6% p<0.0001 |
| [Alanee and Shukla 2009](#_ENREF_2) | USA | SEER, 21 reg. | 1973-2005  (13 reg.) | 195 | Testicular |  | 0.5% [-2.0;1.0] |
| [Poynter, et al. 2010](#_ENREF_51) | USA | SEER, 9 reg. | 1975-2006 | 1,140 970 | Xb;Xc  Boys  Girls | 0-19 y.o. | 0-9 y.o.: -0.3% [-1.9;1.5]; 10-19 y.o. 1.2% [0.4;2.1] 0-9 y.o. 1.9% [0.3;2.5]; 10-19 y.o. -0.1% [-0.8;0.7] < 10 cases/year |

^(a)^ The first part of the table includes the results from 1) studies based on a recent time period (starting in 1990 or after), and 2) studies based on a time period starting before 1990, with consideration of temporal variations in the most recent years (mainly with nonlinear modeling). The second part of the table includes the studies that considered a log-linear temporal trend on a long time period starting before 1990. Improvements in diagnostic methods and cancer registration have certainly played a role in the increases reported in the latter studies (cf discussion section in the manuscript). ^(b)^ Reg.: registry; ACCIS: Automated Cancer Information System; APCR: Australian Paediatric Cancer Registry; CCR: Canadian Cancer Registry; CCRP: Childhood Cancer Registry of Piedmont; CDC: Centers for Disease Control and Prevention; GCCR: German Childhood Cancer Registry; NPCR: National Program of Cancer Registries; NRCT: National Registry of Childhood Tumors; SCR: Swedish Cancer Registry; SEER: Surveillance, Epidemiology, and Results Program; TCR: Taiwan Cancer Registry. ^(c)^ period considered for time-trend; ^(d)^ Number of cases (N) and diagnostic group (0-14 y.o. if not otherwise specified); n.a.: not available; Xa. Intracranial and intraspinal GCT; Xb. Malignant extracranial and extragonadal GCT; Xc. Malignant gonadal GCT. ^(e)^ Nonlinear variation was considered;

AAPC: Average Annual Percent Change; NS: Not Significant according to the authors

**Supplementary table 11** Spatial variation in the incidence of childhood cancer - main results of the papers published since 2000

| **Reference** | **Country** | **Source of data**^(a)^ | **Period** | **Method**^(b)^ | | **Geographic**  **scale**^(c)^ |  | **N^(d)^** |  | **Main results**^(e)^ |
| --- | --- | --- | --- | --- | --- | --- | --- | --- | --- | --- |
| **Leukemias (since the review by McNally et al. 2004)** | | | | | | | | | | |
| [Selvin, et al. 2004](#_ENREF_58) | USA, SF Bay | NCCLS | 1995-1999 | Clustering | NN dist.  (~CE) | 5 counties (point data) | 112  221 | AL  Controls |  | NS overall NS in all counties except San Francisco county (p=0.01, with 4 cases) |
| [Bellec, et al. 2006](#_ENREF_7) | France | RNHE reg. | 1990-2000 | Clustering  Cluster | PW  Moran’s I  Rogerson SaTScan | 36,565 municip. 3,687 cantons | 4,897 | AL |  | NS overall 1990-1994, 0-14 y.o.: $\hat{b}$=0.012 p=0.06; I=0.006 p=0.04; R: p=0.06 |
| [Wheeler 2007](#_ENREF_71) | USA, Ohio | OCISS  OVS | 1996-2003 | Clustering  Cluster | K-function  CE  SaTScan |  | 738 | AL |  | Clustering: NS Cluster detection: "The findings […] analyses are not consistent for the different cluster detection techniques" |
| [McNally, et al. 2009](#_ENREF_38) | Great Britain | NRCT reg. | 1969-1993 | Clustering | PW | 10,444  wards | 10,737  8,687  1,737 | AL  ALL  ANLL |  | AL  $\hat{b}$=0.045 p=0.004 (0-14 y.o.)  $\hat{b}$=0.03 p=0.02 (1-4 y.o.);  $\hat{b}$=-0.8% NS (5-14 y.o.)  ALL  $\hat{b}$=0.05 p=0.002 (0-14 y.o.)  $\hat{b}$=0.03 p=0.02 (1-4 y.o.)  $\hat{b}$=-0.2% NS (5-14 y.o.)  ANLL: $\hat{b}$=0.00 NS (0-14 y.o.) |
| [Schmiedel, et al. 2010](#_ENREF_56) | Germany | GCCR reg. | 1987-2007 | Clustering  Cluster | Q statistic, PW SaTScan | 12,262 municip. | 11,946 | AL |  | ALL $\hat{b}$=0.003 p=0.39  AML $\hat{b}$=-0.02 p=0.77  "we did not find evidence for clusters, neither overall nor in any of the subgroups" |
| [Kulkarni, et al. 2011](#_ENREF_33) | Canada | ACR reg. | 1982-2004 | Cluster | BN SaTScan | 9 RHA  70 sub-RHA | 525 117 | ALL AML | 0-19 y.o. | "A few potential spatiotemporal clusters were identified. They are likely due to small number of cases and plausibly clinically insignificant." |
| [Schmiedel, et al. 2011](#_ENREF_57) | Denmark | DCR reg. Pop. reg. | 1980-2006 | Mapping  Clustering | Kernel density K-function CE |  | 1,168 ~23,400 | AL  Controls |  | 5 NN of each child (test statistic T):  0-14 AL: NS 2-6 y.o. ALL: T_obs_/T_exp_=1.35 [1.15;1.54] at diag; NS at birth |
| [Demoury, et al. 2012](#_ENREF_14) | France | RNHE reg. | 1990-2006 | Clustering  Cluster | PW  Rogerson SaTScan  FleXScan | 1,895 LZ | 7,675  6,269  1,276 | AL  ALL  AML |  | AL: $\hat{b}$=-0.04 p=0.91; R: p=0.39 one cluster detected over 1990-1994  ALL: $\hat{b}$=-0.04 p=0.90; R: p=0.21 1-4 ALL: $\hat{b}$=-0.05 p=0.93; R: p=0.74 one cluster detected over 1995-2000  AML: $\hat{b}$=-0.004 p=0.90; R: p=0.21 one cluster detected over 1995-2000 |
| [Nyari, et al. 2013](#_ENREF_45) | Hungary, south | HPOG | 1981-1997 | Clustering | PW  Moran’s I | 6 counties  906 settlem. | 134 | ALL | 0-4 y.o. | counties: $\hat{b}$=0.56 p=0.04; boys $\hat{b}$=0.02 p=0.39; girls $\hat{b}$=0.38 p=0.08 settlements: $\hat{b}$=0.68 p=0.0003; boys $\hat{b}$=0.22 p=0.003; girls $\hat{b}$=0.07 p=0.20 Moran: I=0.18 p=0.0012; boys I=0.14 p=0.03; girls I=0.04 p=0.16 Autocorrelation only over 1986-1995 ? |
| [Amin, et al. 2014](#_ENREF_3) | USA, Florida | FAPTP | 2000-2010 | Cluster | SaTScan | ZCTAs | 1,833 | AL |  | purely spatial cluster detected +one multivariate cluster (AL/lymph/CNS) |
| [Ramis, et al. 2015](#_ENREF_52) | Spain, 5 regions | SCCR^1^ reg. Birth Reg. | 1996-2011 | Clustering  Cluster | Point data K-function SaTScan | 5 regions | 1,062 6,372 | AL Controls |  | clustering in one region (<3km) no cluster detected |
| [Jakab, et al. 2017](#_ENREF_24) | Hungary | HCCR reg. | 1991-2015 | Clustering | Moran's I  Geary's C | 3152 municip. | 1,676 | AL+MDS |  | I=-0.01 p=0.20; C=0.84 p=0.76 |
| [Konstantinoudis, et al. 2017](#_ENREF_30) | Switzerland | SCCR^2^ reg. | 1985-2016 | Clustering  Cluster | K-function  CE  Tango's  SaTScan  + global test | point data | 1,865 1,485 272  1,297 1,042 180 | AL ALL AML  AL ALL AML | (Diag)  0-15 y.o.  (Birth) | Overall NS (p=0.52 for birth, p=0.51 for diag) One small cluster detected |
| **Lymphomas** | | | | | | | | | | |
| [McNally, et al. 2003a](#_ENREF_39) | UK, Manchester | MCTR reg. | 1976-2000 | Clustering | PW | ~520 wards | 99 111 | HL NHL |  | PW p=n.a. (1976-1985), p=0.04 (1986-2000) PW p=0.67 (1976-1985), p=0.56 (1986-2000) |
| [McNally, et al. 2009](#_ENREF_38) | Great Britain | NRCT reg. | 1969-1993 | Clustering | PW | 10,444  wards | 3,308  1,364  1,678 | HL  NHL |  | $\hat{b}$=0.007 NS  0-14 y.o. $\hat{b}$=0.007 NS 0-9 y.o. $\hat{b}$=-0.015 NS  0-14 y.o. $\hat{b}$=0.01 NS 0-9 y.o. $\hat{b}$=0.02 NS |
| [Kulkarni, et al. 2011](#_ENREF_33) | Canada | ACR reg. | 1982-2004 | Cluster | BN SaTScan | 9 RHA  70 sub-RHA | 257  111 | HL  NHL | 0-19 y.o. | A few potential spatiotemporal clusters were identified. They are likely due to small number of cases and plausibly clinically insignificant. |
| [Amin, et al. 2014](#_ENREF_3) | USA, Florida | FAPTP | 2000-2010 | Cluster | SaTScan | ZCTAs | 745 | Lymph |  | 1 purely spatial cluster detected +one multivariate cluster (AL/lymph/CNS) |
| [Ramis, et al. 2015](#_ENREF_52) | Spain, 5 regions | SCCR^1^  Birth Reg. | 1996-2011 | Clustering  Cluster | K-function Diggle SaTScan | 5 regions  Point data | 92  (552) 246  (1,476) | HL  (controls) NHL  (controls) |  | clustering in one region (2-6km) no cluster detected |
| **CNS tumors** | | | | | | | | | | |
| [McNally, et al. 2004](#_ENREF_41) | UK, Manchester | MCTR reg. | 1976-2000 | Clustering | PW  Swans | 519 wards  24 districts | 567  229  47  93  79 | CNS tum.  Astrocyt.  Ependym.  Medullo.  Other glio. |  | PW:"p=0.10 to 0.67", Swans "p=0.25 to 0.66"  PW:"p=0.40 to 0.82", Swans "p=0.36 to 0.95"  NS  PW: "p=0.03 on 1985-2000"  NS |
| [McNally, et al. 2009](#_ENREF_38) | Great Britain | NRCT reg. | 1969-1993 | Clustering | PW | 10,444  wards | 7,473 | CNS tum. |  | "$\hat{b}$=0.001 NS" |
| [Rosychuk, et al. 2010](#_ENREF_54) | Canada | ACR reg. | 1982-2004 | Cluster | BN SaTScan ST | 9 RHA  70 sub-RHA | 568 | CNS tum. | 0-19 y.o. | "Our results did not identify areas that were both statistically and clinically significant." |
| [Amin, et al. 2014](#_ENREF_3) | USA, Florida | FAPTP | 2000-2010 | Cluster | SaTScan | ZCTAs | 1,194 | CNS+  brain |  | purely spatial cluster detected +one multivariate cluster (AL/lymph/CNS) |
| [Ramis, et al. 2015](#_ENREF_52) | Spain, 5 regions | SCCR^1^  Birth Reg. | 1996-2011 | Clustering  Cluster | K-function Diggle SaTScan | 5 regions (Point data) | 714 4,284 | CNS Controls |  | clustering in one region (<1km) no cluster detected |
| **Other solid tumors** | | | | | | | | | | |
| [McNally, et al. 2003b](#_ENREF_40) | UK, Manchester | MCTR reg. | 1976-2000 | Clustering | PW  Swans | ~520 wards  24 districts | 120 | Wilm's tum. |  | PW: p=0.06 (district) PW: p=0.01 (wards 1976-85, 70 cases)  Swans: NS |
| [McNally, et al. 2009](#_ENREF_38) | Great Britain | NRCT reg. | 1969-1993 | Clustering | PW | 10,444  wards | 1,890 | Renal tum. |  | $\hat{b}$=0.04 p=0.007 |
| [McNally, et al. 2003a](#_ENREF_39) | UK, Manchester | MCTR reg. | 1976-2000 | Clustering | PW Swans | ~520 wards  24 districts | 125  75 | STS  RMS |  | PW: p=0.79 (districts) NS (wards) Swans: p=0.09 (wards 1976-1985, 79 cases)  NS |
| [McNally, et al. 2009](#_ENREF_38) | Great Britain | NRCT reg. | 1969-1993 | Clustering | PW | 10,444  wards | 2,101 | STS |  | $\hat{b}$=0.03 p=0.04 |
|  | Great Britain | NRCT reg. | 1969-1993 | Clustering | PW | 10,444  wards | 2,111  1,507  983 | SNS tum.  Bone tum.  GCT |  | $\hat{b}$=-0.02 NS  $\hat{b}$=0.008 NS  $\hat{b}$=0.004 NS |

^(a)^ reg.: registry; ACR: Alberta Cancer Registry; CPR: Central Population Registry; DCR: Danish Cancer Registry; FAPTP: Florida Association for Pediatric Tumor Programs; GCCR: German Childhood Cancer Registry; HCCR: Hungarian Childhood Cancer Registry; HPOG: Hungarian Paediatric Oncology Group; MCTR: Manchester Childhood Tumors Registry; NCCLS: Northern California Childhood Leukemia Study; NRCT: National Registry of Childhood Tumors; OCISS: Ohio Cancer Incidence Surveillance System; OVS: Ohio Vital Statistics; RHA: Regional Health Authorities areas; RNHE: National registry of childhood hematological malignancies; SCCR1: Spanish Childhood Cancer Registry; SCCR2: Swiss Childhood Cancer Registry. ^(b)^ Cluster: cluster detection method; clustering: test for global heterogeneity; BN: Besag and Newell’s test for cluster detection; CE: Cuzick and Edward’s test for spatial clustering; PW: Potthoff and Whittinghill test for over-dispersion; ^(c)^ municip.: municipalities; LZ: living zone; RHA: regional Health authorities; settlem.: settlement; ZCTA: ZIP Code Tabulation Areas. ^(d)^ N: Number of cases (and controls if applicable) and diagnostic group (0-14 y.o. if not otherwise specified); AL: acute leukemia; ALL: acute lymphoid leukemia; AML: acute myeloid leukemia; ANLL: acute non lymphoid leukemia; CNS tum.: central nervous system tumor; GCT: germ cell tumor; HL: Hodgkin lymphoma; MDS: myelodysplastic syndrome; NHL: non-Hodgkin lymphoma; RMS: rhabdomyosarcoma; STS: soft tissues sarcoma; tum.: tumor. ^(e)^ $\hat{b}$: over-dispersion parameter estimate; NS: not significant result according to the authors

**Supplementary table 12** Distribution of the person-years at risk by year of age from 2000 to 2014, mainland France (column percentages)

| Age | 2000 | 2001 | 2002 | 2003 | 2004 | 2005 | 2006 | 2007 | 2008 | 2009 | 2010 | 2011 | 2012 | 2013 | 2014 |
| --- | --- | --- | --- | --- | --- | --- | --- | --- | --- | --- | --- | --- | --- | --- | --- |
| 0 | 7% | 7% | 7% | 7% | 7% | 7% | 7% | 7% | 7% | 7% | 7% | 7% | 7% | 6% | 6% |
| 1 | 7% | 7% | 7% | 7% | 7% | 7% | 7% | 7% | 7% | 7% | 7% | 7% | 7% | 7% | 6% |
| 2 | 6% | 7% | 7% | 7% | 7% | 7% | 7% | 7% | 7% | 7% | 7% | 7% | 7% | 7% | 7% |
| 3 | 6% | 6% | 7% | 7% | 7% | 7% | 7% | 7% | 7% | 7% | 7% | 7% | 7% | 7% | 7% |
| 4 | 6% | 6% | 7% | 7% | 7% | 7% | 7% | 7% | 7% | 7% | 7% | 7% | 7% | 7% | 7% |
| 5 | 6% | 6% | 7% | 7% | 7% | 7% | 7% | 7% | 7% | 7% | 7% | 7% | 7% | 7% | 7% |
| 6 | 6% | 6% | 7% | 7% | 7% | 7% | 7% | 7% | 7% | 7% | 7% | 7% | 7% | 7% | 7% |
| 7 | 6% | 6% | 6% | 7% | 7% | 7% | 7% | 7% | 7% | 7% | 7% | 7% | 7% | 7% | 7% |
| 8 | 7% | 6% | 6% | 6% | 7% | 7% | 7% | 7% | 7% | 7% | 7% | 7% | 7% | 7% | 7% |
| 9 | 7% | 7% | 6% | 6% | 6% | 7% | 7% | 7% | 7% | 7% | 7% | 7% | 7% | 7% | 7% |
| 10 | 7% | 7% | 7% | 7% | 6% | 7% | 7% | 7% | 7% | 7% | 7% | 7% | 7% | 7% | 7% |
| 11 | 7% | 7% | 7% | 7% | 7% | 6% | 7% | 7% | 7% | 7% | 7% | 7% | 7% | 7% | 7% |
| 12 | 7% | 7% | 7% | 7% | 7% | 7% | 6% | 7% | 7% | 7% | 7% | 7% | 7% | 7% | 7% |
| 13 | 7% | 7% | 7% | 7% | 7% | 7% | 7% | 6% | 7% | 7% | 7% | 7% | 7% | 7% | 7% |
| 14 | 7% | 7% | 7% | 7% | 7% | 7% | 7% | 7% | 6% | 6% | 7% | 7% | 7% | 7% | 7% |
| 0-14 PY* | 11.1 | 11.2 | 11.2 | 11.2 | 11.2 | 11.2 | 11.3 | 11.3 | 11.4 | 11.5 | 11.6 | 11.6 | 11.7 | 11.8 | 11.8 |
|  | (100%) | (100%) | (100%) | (100%) | (100%) | (100%) | (100%) | (100%) | (100%) | (100%) | (100%) | (100%) | (100%) | (100%) | (100%) |

*: person-years at risk in million children estimated by mid-year populations

**Supplementary figure 1** Annual incidence rate of childhood Burkitt lymphomas (and 95% CI) between 2000 and 2014 and estimated log-linear trend (dashed line)

**Supplementary figure 2** Annual incidence rate (and 95% CI) of childhood malignant germ-cell tumors between 2000 and 2014 and estimated log-linear trend (dashed line)

**References**

Agha M, DiMonte B, Greenberg M, Greenberg C, Barr R, McLaughlin JR. Incidence trends and projections for childhood cancer in Ontario. Int J Cancer 2006;**118**: 2809-15.

Alanee S, Shukla A. Paediatric testicular cancer: an updated review of incidence and conditional survival from the Surveillance, Epidemiology and End Results database. BJU Int 2009;**104**: 1280-3.

Amin R, Hendryx M, Shull M, Bohnert A. A cluster analysis of Pediatric Cancer Incidence Rates in Florida: 2000-2010. Statistics and Public Policy 2014;**1**: 69-77.

Baade PD, Youlden DR, Valery PC, Hassall T, Ward L, Green AC, Aitken JF. Trends in incidence of childhood cancer in Australia, 1983-2006. Br J Cancer 2010;**102**: 620-6.

Bao PP, Zheng Y, Gu K, Wang CF, Wu CX, Jin F, Lu W. Trends in childhood cancer incidence and mortality in urban Shanghai, 1973-2005. Pediatric Blood & Cancer 2010;**54**: 1009-13.

Barrington-Trimis JL, Cockburn M, Metayer C, Gauderman WJ, Wiemels J, McKean-Cowdin R. Trends in childhood leukemia incidence over two decades from 1992 to 2013. Int J Cancer 2017;**140**: 1000-8.

Bellec S, Hemon D, Rudant J, Goubin A, Clavel J. Spatial and space-time clustering of childhood acute leukaemia in France from 1990 to 2000: a nationwide study. Br J Cancer 2006;**94**: 763-70.

Broaddus E, Topham A, Singh AD. Incidence of retinoblastoma in the USA: 1975-2004. Br J Ophthalmol 2009;**93**: 21-3.

Clavel J, Goubin A, Auclerc MF, Auvrignon A, Waterkeyn C, Patte C, Baruchel A, Leverger G, Nelken B, Philippe N, Sommelet D, Vilmer E, et al. Incidence of childhood leukaemia and non-Hodgkin's lymphoma in France: National Registry of Childhood Leukaemia and Lymphoma, 1990-1999. Eur J Cancer Prev 2004;**13**: 97-103.

Clavel J, Steliarova-Foucher E, Berger C, Danon S, Valerianova Z. Hodgkin's disease incidence and survival in European children and adolescents (1978-1997): report from the Automated Cancer Information System project. Eur J Cancer 2006;**42**: 2037-49.

Cotterill SJ, Parker L, Malcolm AJ, Reid M, More L, Craft AW. Incidence and survival for cancer in children and young adults in the North of England, 1968-1995: a report from the Northern Region Young Persons' Malignant Disease Registry. Br J Cancer 2000;**83**: 397-403.

Dalmasso P, Pastore G, Zuccolo L, Maule MM, Pearce N, Merletti F, Magnani C. Temporal trends in the incidence of childhood leukemia, lymphomas and solid tumors in north-west Italy, 1967-2001. A report of the Childhood Cancer Registry of Piedmont. Haematologica 2005;**90**: 1197-204.

Demanelis K, Sriplung H, Meza R, Wiangnon S, Rozek LS, Scheurer ME, Lupo PJ. Differences in childhood leukemia incidence and survival between Southern Thailand and the United States: a population-based analysis. Pediatric Blood & Cancer 2015;**62**: 1790-8.

Demoury C, Goujon-Bellec S, Guyot-Goubin A, Hemon D, Clavel J. Spatial variations of childhood acute leukaemia in France, 1990-2006: global spatial heterogeneity and cluster detection at 'living-zone' level. Eur J Cancer Prev 2012;**21**: 367-74.

Desandes E, Guissou S, Chastagner P, Lacour B. Incidence and survival of children with central nervous system primitive tumors in the French National Registry of Childhood Solid Tumors. Neuro Oncol 2014;**16**: 975-83.

Dreifaldt AC, Carlberg M, Hardell L. Increasing incidence rates of childhood malignant diseases in Sweden during the period 1960-1998. Eur J Cancer 2004;**40**: 1351-60.

Gittleman HR, Ostrom QT, Rouse CD, Dowling JA, de Blank PM, Kruchko CA, Elder JB, Rosenfeld SS, Selman WR, Sloan AE, Barnholtz-Sloan JS. Trends in central nervous system tumor incidence relative to other common cancers in adults, adolescents, and children in the United States, 2000 to 2010. Cancer 2015;**121**: 102-12.

Goujon-Bellec S, Mollie A, Rudant J, Guyot-Goubin A, Clavel J. Time trends and seasonal variations in the diagnosis of childhood acute lymphoblastic leukaemia in France. Cancer Epidemiol 2013;**37**: 255-61.

Hjalgrim LL, Rostgaard K, Engholm G, Pukkala E, Johannesen TB, Olafsdottir E, Hjalgrim H. Aetiologic heterogeneity in pediatric Hodgkin lymphoma? Evidence from the Nordic countries, 1978-2010. Acta Oncol 2016;**55**: 85-90.

Hung GY, Horng JL, Lee YS, Yen HJ, Chen CC, Lee CY. Cancer incidence patterns among children and adolescents in Taiwan from 1995 to 2009: a population-based study. Cancer 2014a;**120**: 3545-53.

Hung GY, Horng JL, Yen HJ, Yen CC, Chen WM, Chen PC, Wu HT, Chiou HJ. Incidence patterns of primary bone cancer in taiwan (2003-2010): a population-based study. Ann Surg Oncol 2014b;**21**: 2490-8.

Isaevska E, Manasievska M, Alessi D, Mosso ML, Magnani C, Sacerdote C, Pastore G, Fagioli F, Merletti F, Maule M. Cancer incidence rates and trends among children and adolescents in Piedmont, 1967-2011. PLoS One 2017;**12**: e0181805.

Izarzugaza MI, Steliarova-Foucher E, Martos MC, Zivkovic S. Non-Hodgkin's lymphoma incidence and survival in European children and adolescents (1978-1997): report from the Automated Childhood Cancer Information System project. Eur J Cancer 2006;**42**: 2050-63.

Jakab Z, Juhasz A, Nagy C, Schuler D, Garami M. Trends and territorial inequalities of incidence and survival of childhood leukaemia and their relations to socioeconomic status in Hungary, 1971-2015. Eur J Cancer Prev 2017;**26 Joining forces for better cancer registration in Europe**: S183-S90.

Johannesen TB, Angell-Andersen E, Tretli S, Langmark F, Lote K. Trends in incidence of brain and central nervous system tumors in Norway, 1970-1999. Neuroepidemiology 2004;**23**: 101-9.

Kaatsch P, Hafner C, Calaminus G, Blettner M, Tulla M. Pediatric germ cell tumors from 1987 to 2011: incidence rates, time trends, and survival. Pediatrics 2015;**135**: e136-43.

Kaatsch P, Mergenthaler A. Incidence, time trends and regional variation of childhood leukaemia in Germany and Europe. Radiat Prot Dosimetry 2008;**132**: 107-13.

Kaatsch P, Steliarova-Foucher E, Crocetti E, Magnani C, Spix C, Zambon P. Time trends of cancer incidence in European children (1978-1997): report from the Automated Childhood Cancer Information System project. Eur J Cancer 2006;**42**: 1961-71.

Kamsa-ard S, Wiangnon S, Suwanrungruang K, Jetsrisuparb A, Horsith S. Trends in incidence of childhood leukemia, Khon Kaen, Thailand, 1985-2002. Asian Pac J Cancer Prev 2006;**7**: 75-8.

Konstantinoudis G, Kreis C, Ammann RA, Niggli F, Kuehni CE, Spycher BD. Spatial clustering of childhood leukaemia in Switzerland: A nationwide study. Int J Cancer 2017;**141**: 1324-32.

Kroll ME, Carpenter LM, Murphy MF, Stiller CA. Effects of changes in diagnosis and registration on time trends in recorded childhood cancer incidence in Great Britain. Br J Cancer 2012;**107**: 1159-62.

Kroll ME, Draper GJ, Stiller CA, Murphy MF. Childhood leukemia incidence in Britain, 1974-2000: time trends and possible relation to influenza epidemics. J Natl Cancer Inst 2006;**98**: 417-20.

Kulkarni K, Stobart K, Witol A, Rosychuk RJ. Leukemia and lymphoma incidence in children in Alberta, Canada: a population-based 22-year retrospective study. Pediatr Hematol Oncol 2011;**28**: 649-60.

Linabery AM, Ross JA. Trends in childhood cancer incidence in the U.S. (1992-2004). Cancer 2008;**112**: 416-32.

Linet MS, Brown LM, Mbulaiteye SM, Check D, Ostroumova E, Landgren A, Devesa SS. International long-term trends and recent patterns in the incidence of leukemias and lymphomas among children and adolescents ages 0-19 years. Int J Cancer 2016;**138**: 1862-74.

Maule MM, Zuccolo L, Magnani C, Pastore G, Dalmasso P, Pearce N, Merletti F, Gregori D. Bayesian methods for early detection of changes in childhood cancer incidence: trends for acute lymphoblastic leukaemia are consistent with an infectious aetiology. Eur J Cancer 2006;**42**: 78-83.

McKean-Cowdin R, Razavi P, Barrington-Trimis J, Baldwin RT, Asgharzadeh S, Cockburn M, Tihan T, Preston-Martin S. Trends in childhood brain tumor incidence, 1973-2009. J Neurooncol 2013;**115**: 153-60.

McNally RJ, Alexander FE, Vincent TJ, Murphy MF. Spatial clustering of childhood cancer in Great Britain during the period 1969-1993. Int J Cancer 2009;**124**: 932-6.

McNally RJ, Alston RD, Cairns DP, Eden OB, Birch JM. Geographical and ecological analyses of childhood acute leukaemias and lymphomas in north-west England. Br J Haematol 2003a;**123**: 60-5.

McNally RJ, Alston RD, Cairns DP, Eden OB, Kelsey AM, Birch JM. Geographical and ecological analyses of childhood Wilms' tumours and soft-tissue sarcomas in North West England. Eur J Cancer 2003b;**39**: 1586-93.

McNally RJ, Alston RD, Eden TO, Kelsey AM, Birch JM. Further clues concerning the aetiology of childhood central nervous system tumours. Eur J Cancer 2004;**40**: 2766-72.

McNally RJ, Cairns DP, Eden OB, Kelsey AM, Taylor GM, Birch JM. Examination of temporal trends in the incidence of childhood leukaemias and lymphomas provides aetiological clues. Leukemia 2001;**15**: 1612-8.

McNeil DE, Cote TR, Clegg L, Rorke LB. Incidence and trends in pediatric malignancies medulloblastoma/primitive neuroectodermal tumor: a SEER update. Surveillance Epidemiology and End Results. Med Pediatr Oncol 2002;**39**: 190-4.

Mitra D, Shaw AK, Hutchings K. Trends in incidence of childhood cancer in Canada, 1992-2006. Chronic Dis Inj Can 2012;**32**: 131-9.

Nyari TA, Ottoffy G, Bartyik K, Thurzo L, Solymosi N, Cserni G, Parker L, McNally RJ. Spatial clustering of childhood acute lymphoblastic leukaemia in hungary. Pathol Oncol Res 2013;**19**: 297-302.

Ognjanovic S, Linabery AM, Charbonneau B, Ross JA. Trends in childhood rhabdomyosarcoma incidence and survival in the United States, 1975-2005. Cancer 2009;**115**: 4218-26.

Papathoma P, Thomopoulos TP, Karalexi MA, Ryzhov A, Zborovskaya A, Dimitrova N, Zivkovic S, Eser S, Antunes L, Sekerija M, Zagar T, Bastos J, et al. Childhood central nervous system tumours: Incidence and time trends in 13 Southern and Eastern European cancer registries. Eur J Cancer 2015;**51**: 1444-55.

Pastore G, Peris-Bonet R, Carli M, Martinez-Garcia C, Sanchez de Toledo J, Steliarova-Foucher E. Childhood soft tissue sarcomas incidence and survival in European children (1978-1997): report from the Automated Childhood Cancer Information System project. Eur J Cancer 2006;**42**: 2136-49.

Peris-Bonet R, Salmeron D, Martinez-Beneito MA, Galceran J, Marcos-Gragera R, Felipe S, Gonzalez V, Sanchez de Toledo Codina J. Childhood cancer incidence and survival in Spain. Ann Oncol 2010;**21 Suppl 3**: iii103-10.

Petridou ET, Dimitrova N, Eser S, Kachanov D, Karakilinc H, Varfolomeeva S, Belechri M, Baka M, Moschovi M, Polychronopoulou S, Athanasiadou-Piperopoulou F, Fragandrea I, et al. Childhood leukemia and lymphoma: time trends and factors affecting survival in five Southern and Eastern European Cancer Registries. Cancer Causes Control 2013;**24**: 1111-8.

Poynter JN, Amatruda JF, Ross JA. Trends in incidence and survival of pediatric and adolescent patients with germ cell tumors in the United States, 1975 to 2006. Cancer 2010;**116**: 4882-91.

Ramis R, Gomez-Barroso D, Tamayo I, Garcia-Perez J, Morales A, Pardo Romaguera E, Lopez-Abente G. Spatial analysis of childhood cancer: a case/control study. PLoS One 2015;**10**: e0127273.

Rendon-Macias ME, Valencia-Ramon EA, Fajardo-Gutierrez A, Rivera-Flores E. Childhood lymphoma incidence patterns by ICCC-3 subtype in Mexico City metropolitan area population insured by Instituto Mexicano del Seguro Social, 1996-2010. Cancer Causes Control 2015;**26**: 849-57.

Rosychuk RJ, Witol A, Stobart K. Childhood cancer trends in a western Canadian province: a population-based 22-year retrospective study. Pediatric Blood & Cancer 2010;**55**: 1348-55.

Schmidt LS, Schmiegelow K, Lahteenmaki P, Trager C, Stokland T, Grell K, Gustafson G, Sehested A, Raashou-Nielsen O, Johansen C, Schuz J. Incidence of childhood central nervous system tumors in the Nordic countries. Pediatric Blood & Cancer 2011;**56**: 65-9.

Schmiedel S, Blettner M, Kaatsch P, Schuz J. Spatial clustering and space-time clusters of leukemia among children in Germany, 1987-2007. Eur J Epidemiol 2010;**25**: 627-33.

Schmiedel S, Jacquez GM, Blettner M, Schuz J. Spatial clustering of leukemia and type 1 diabetes in children in Denmark. Cancer Causes Control 2011;**22**: 849-57.

Selvin S, Ragland KE, Chien EY, Buffler PA. Spatial analysis of childhood leukemia in a case/control study. Int J Hyg Environ Health 2004;**207**: 555-62.

Seregard S, Lundell G, Svedberg H, Kivela T. Incidence of retinoblastoma from 1958 to 1998 in Northern Europe: advantages of birth cohort analysis. Ophthalmology 2004;**111**: 1228-32.

Shah A, Coleman MP. Increasing incidence of childhood leukaemia: a controversy re-examined. Br J Cancer 2007;**97**: 1009-12.

Siegel DA, King J, Tai E, Buchanan N, Ajani UA, Li J. Cancer incidence rates and trends among children and adolescents in the United States, 2001-2009. Pediatrics 2014;**134**: e945-55.

Spix C, Eletr D, Blettner M, Kaatsch P. Temporal trends in the incidence rate of childhood cancer in Germany 1987-2004. Int J Cancer 2008;**122**: 1859-67.

Srina A, Jetsrisuparb A, Komvilaisak P, Kamsaard S, Wiangnon S. Trends in incidence of childhood lymphoma in Khon Kaen, Thailand, 1985-2008. Asian Pac J Cancer Prev 2010;**11**: 1683-6.

Steliarova-Foucher E, Stiller C, Kaatsch P, Berrino F, Coebergh JW, Lacour B, Parkin M. Geographical patterns and time trends of cancer incidence and survival among children and adolescents in Europe since the 1970s (the ACCISproject): an epidemiological study. Lancet 2004;**364**: 2097-105.

Stiller CA, Bielack SS, Jundt G, Steliarova-Foucher E. Bone tumours in European children and adolescents, 1978-1997. Report from the Automated Childhood Cancer Information System project. Eur J Cancer 2006;**42**: 2124-35.

Svendsen AL, Feychting M, Klaeboe L, Langmark F, Schuz J. Time trends in the incidence of acute lymphoblastic leukemia among children 1976-2002: a population-based Nordic study. J Pediatr 2007;**151**: 548-50.

Tulla M, Berthold F, Graf N, Rutkowski S, von Schweinitz D, Spix C, Kaatsch P. Incidence, Trends, and Survival of Children With Embryonal Tumors. Pediatrics 2015;**136**: e623-32.

Walsh TJ, Grady RW, Porter MP, Lin DW, Weiss NS. Incidence of testicular germ cell cancers in U.S. children: SEER program experience 1973 to 2000. Urology 2006;**68**: 402-5; discussion 5.

Ward E, DeSantis C, Robbins A, Kohler B, Jemal A. Childhood and adolescent cancer statistics, 2014. CA Cancer J Clin 2014;**64**: 83-103.

Weihkopf T, Blettner M, Dantonello T, Jung I, Klingebiel T, Koscielniak E, Luckel M, Spix C, Kaatsch P. Incidence and time trends of soft tissue sarcomas in German children 1985-2004 - a report from the population-based German Childhood Cancer Registry. Eur J Cancer 2008;**44**: 432-40.

Wheeler DC. A comparison of spatial clustering and cluster detection techniques for childhood leukemia incidence in Ohio, 1996-2003. Int J Health Geogr 2007;**6**: 13.
